# A Physics-Guided Graph Transformer for Short-Term SCADA-Based Multi-Turbine Wind Power Forecasting

**DOI:** 10.3390/s26216672

**Published:** 2026-10-22

**Authors:** Weicheng Hu, Chunhua Hu

**Affiliations:** 1 College of Materials Science and Engineering, Nanjing Forestry University, Nanjing 210037, China; 2 College of Information Science and Technology & College of Artificial Intelligence, Nanjing Forestry University, No. 159 Longpan Road, Nanjing 210037, China

**Keywords:** wind power forecasting, graph neural networks, physics-guided learning, Transformer, probabilistic forecasting, SCADA data, uncertainty quantification

## Abstract

Short-term wind power forecasting depends on reliable Supervisory Control and Data Acquisition (SCADA) measurements and on models that can represent interactions across a turbine fleet. PG-RAGT is proposed for this setting as a physics-guided graph Transformer for 10–60 min multi-turbine power forecasting. The contribution is organized at three levels: application-oriented system integration, state-conditioned fleet interaction, and forecast-time physical-state closure. Physics-Consistent SCADA Screening acts as a nominal-regime training filter for active-power observations that depart strongly from a simplified aerodynamic reference. Axial Fleet Blocks form sample- and patch-dependent cross-turbine interaction matrices from the current SCADA representation without a predefined adjacency. Gated Physical-State Rolling generates horizon-dependent wind speed, external temperature, and mean pitch angle from the last observed state, allowing a state-dependent aerodynamic power prior to be evaluated without future SCADA measurements. On the SDWPF test set, the main seed gives RMSE = 106.69 kW, *R*^2^ = 0.9125, and MAE = 57.12 kW; across three independently seeded runs, the mean RMSE is 107.19 kW. The cross-turbine pathway shows the most consistent ablation effect, whereas the screening and rolling effects are smaller and more training-run dependent. The resulting framework provides comparatively accurate fleet-scale forecasts while retaining probabilistic outputs and a reduced-model option for lower computational cost.

## 1. Introduction

Wind power forecasting supports grid dispatch and wind farm operation by providing advance estimates of future power output. Forecast accuracy depends not only on recent power history but also on the current operating state of each turbine. From a sensing perspective, a wind farm can be viewed as a distributed multi-sensor system in which Supervisory Control and Data Acquisition (SCADA) measurements from multiple turbines must be screened and fused for short-term forecasting. The literature has shifted from physical and statistical methods toward machine learning and deep learning [1,2,3], and studies using high-frequency SCADA measurements show that explicit temporal modeling can improve short-term prediction [4].

The predictive value of such SCADA-based modeling depends first on input reliability. In practice, SCADA records may contain non-nominal operating data associated with curtailment or turbine shutdowns, as well as missing or corrupted measurements caused by sensor and communication faults. Consequently, the recorded data may be incomplete or may deviate from the aerodynamic relationships expected during nominal operation. Data-quality assessment should therefore be treated as part of the forecasting pipeline rather than as an isolated preprocessing step [5,6]. Recent industrial sensor-quality work has also combined anomaly detection, fault localization, and data reconstruction [7]; the screening considered here is narrower and does not infer sensor-fault classes or reconstruct channels. Power-curve cleaning studies similarly show that training on aerodynamically inconsistent observations can distort the learned power mapping [8,9].

After data-quality control, forecasting models must capture temporal dependencies in the cleaned SCADA sequences [10]. To alleviate the vanishing-gradient problem in conventional recurrent neural networks (RNNs), Long Short-Term Memory (LSTM) networks [11] and Gated Recurrent Units (GRUs) [12] employ gating mechanisms, with GRUs generally using a simpler architecture and lower computational complexity. However, LSTM and GRU models may still have difficulty capturing long-range dependencies. In 2017, Vaswani et al. introduced the Transformer architecture, which uses self-attention to model global dependencies and preserve contextual information [13]. For long time-series inputs, Informer and PatchTST employ sparse attention and patch-based segmentation, respectively, to reduce computational cost [14,15]. Recent industrial time-series models have likewise combined temporal convolution, recurrent modeling, channel attention, and multi-scale extraction [16], so multi-scale extraction is treated here as an established building block rather than a new operator. However, these methods primarily model temporal dependencies within individual turbine sequences and do not explicitly capture interactions among turbines. Since multiple turbines are observed simultaneously and their power outputs can be interdependent, fleet-scale forecasting also requires spatial dependency modeling.

To address this limitation, graph-based methods have been introduced to model relationships among turbines. The Graph Convolutional Network (GCN) proposed by Kipf and Welling and the Graph Attention Network (GAT) introduced by Veličković et al. provide widely used mechanisms for modeling such inter-turbine relationships through graph convolution and attention, respectively [17,18]. In 2020, Bai et al. proposed the Adaptive Graph Convolutional Recurrent Network (AGCRN), which learns adaptive inter-node relationships without requiring a predefined graph structure [19]. Since then, graph-based wind power forecasting methods have increasingly combined SCADA measurements, wind turbine layouts, and time-series models to capture both spatial and temporal dependencies [20,21,22]. In [23], graph attention was combined with LSTM for short-term wind power forecasting. Liu et al. further proposed a spatiotemporal physics-guided graph neural network, in which graph representation learning incorporates the physical interaction mechanisms among wind turbines [24]. Graph attention has therefore become an important approach for modeling dependencies among wind turbines. These developments are particularly relevant to fleet-scale benchmarks such as the SDWPF dataset [25], which contains 10 min SCADA measurements from 134 wind turbines.

Beyond temporal and spatial dependency modeling, recent studies have also incorporated physical knowledge and predictive uncertainty into wind power forecasting. Physics-informed models incorporate physical constraints directly into model training [26], whereas physics-guided methods use physical or meteorological knowledge to support prediction [27]. Point forecasts do not quantify predictive uncertainty, whereas quantile regression provides a practical means of constructing prediction intervals [28,29,30]. Transformer-based models have also incorporated uncertainty estimation into temporal feature learning [31].

Wind-turbine operating conditions can be non-stationary and temporally correlated under variable loading [32]. Against this background, the practical challenge is not a single missing operator but the need to satisfy several requirements simultaneously in a SCADA-only fleet-forecasting setting. First, numerically plausible active-power measurements may still depart from the nominal wind speed–power relationship even after conventional range and missing-value processing. Second, inter-turbine dependencies can vary with operating state and temporal context, so a fixed interaction structure may be restrictive. Third, a state-dependent aerodynamic power model requires future wind speed, external temperature, and mean pitch angle, but these quantities are unavailable as future SCADA measurements at forecast time. Taken together, these gaps motivate an application-oriented system design that can handle training-data consistency, state-dependent fleet interaction, and forecast-time physical guidance within one operational forecasting pipeline.

To address these issues, this study proposes PG-RAGT, a physics-guided graph Transformer for SCADA-based multi-turbine short-term wind power forecasting. The contributions are organized at three levels. (1) At the application/system level, PG-RAGT integrates nominal-regime SCADA screening, temporal–fleet representation, forecast-time physical guidance, reliability-weighted fusion, and uncertainty estimation into a unified SCADA-only 10–60 min forecasting framework. (2) At the architectural level, two Axial Fleet Blocks organize temporal-patch-wise cross-turbine attention to learn sample- and patch-dependent interaction weights without a predefined adjacency matrix, allowing fleet representations to adapt to the current operating context. (3) At the methodological level, Gated Physical-State Rolling converts learned forecast-horizon representations into bounded wind speed, temperature, and pitch estimates from the last observed state. This supplies the missing future physical inputs for horizon-specific aerodynamic-prior evaluation without using future SCADA measurements, a function not provided by adaptive fleet mixing and a static physical prior alone. Physics-Consistent SCADA Screening serves as a supporting training-data filter. Experiments assess forecasting accuracy, component contributions, operating regimes, uncertainty, computational efficiency, and external-site applicability after retraining.

## 2. Materials

### 2.1. Data

#### 2.1.1. SDWPF Dataset

**Figure 1:**
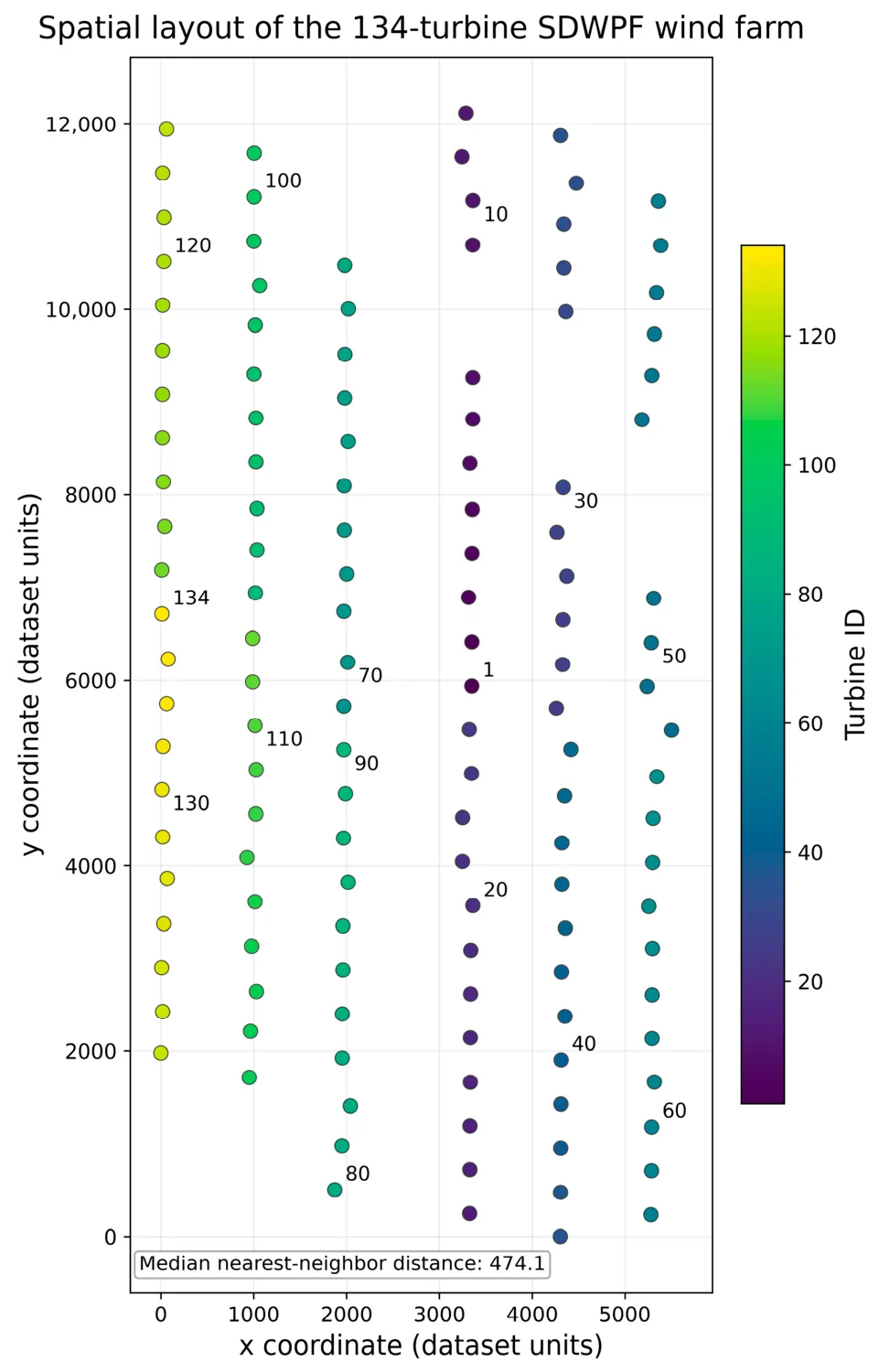
Schematic illustration of the approximate spatial layout of the 134-turbine SDWPF wind farm. The coordinate values are reported in the dataset coordinate units because a verified physical unit is not provided with the layout used here.

The experiments are conducted on the public SDWPF dataset, a benchmark from the 2022 Baidu Knowledge Discovery and Data Mining (KDD) Cup containing 245 days of 10 min SCADA data from 134 1.55 MW wind turbines, with more than 4.7 million records. For forecasting, 144 historical time steps (24 h) are used to predict the subsequent 6 steps (1 h). To prevent data leakage, the dataset is chronologically split into 70% for training, 15% for validation, and 15% for testing. This setting prevents the model from accessing future observations and more closely reflects a realistic industrial forecasting scenario. The spatial layout of the 134 turbines is shown in Figure 1.

#### 2.1.2. Penmanshiel External Dataset

**Figure 2:**
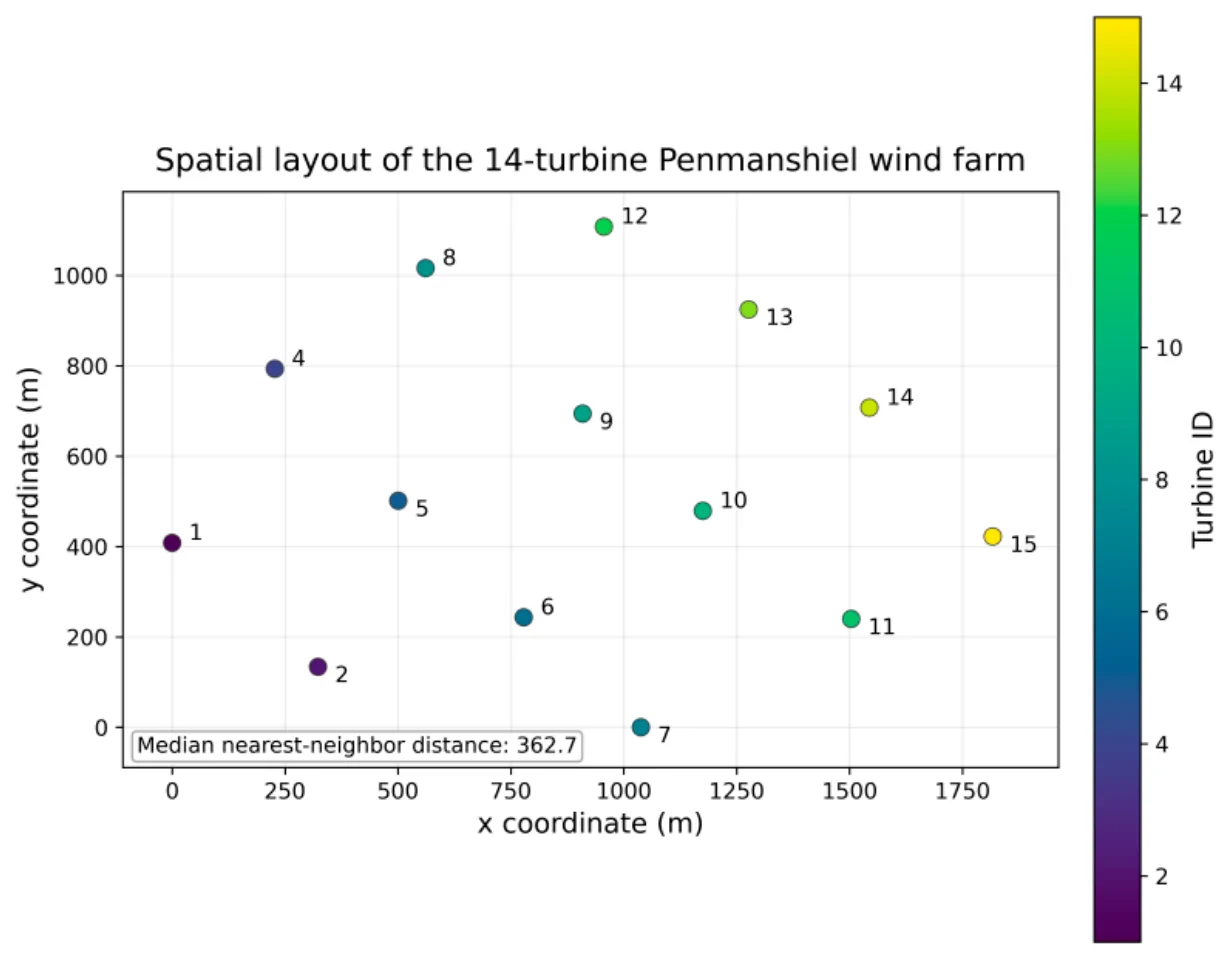
Spatial layout of the 14-turbine Penmanshiel wind farm.

The Penmanshiel dataset [33] is used for external-site validation after retraining. It contains 10 min SCADA records collected in 2017 from 14 Senvion MM82 turbines (Senvion GmbH, Hamburg, Germany; WT01, WT02, and WT04–WT15), each modeled using an adopted power reference of 2000 kW in this study. The Penmanshiel experiments use the same chronological 70%/15%/15% split, 144-step input window, 6-step forecasting horizon, preprocessing sequence, and evaluation metrics as the SDWPF experiments. For this site, the Differentiable Wind Power Prior uses the adopted power reference of 2000 kW and a rotor radius of 41.0 m. Figure 2 shows the spatial layout of the 14 turbines.

### 2.2. Data Processing

Data preprocessing consists of four stages: sentinel-value screening, Physics-Consistent SCADA Screening, missing-value imputation, and range clipping. Sentinel-value screening is first applied to measurements outside the admissible ranges defined for the raw SCADA channels; Physics-Consistent SCADA Screening is then applied to active power. Rather than imposing the same physical criterion over all operating regions, screening is restricted to the adopted interval 3 < wind speed (Wspd) < 12.5 m/s and uses a simplified nominal aerodynamic reference. With the power coefficient *C_p_* = 0.45, rotor radius *R* = 38.5 m, and air density *ρ* = 1.225 kg/m^3^, the normalized residual between measured active power and the nominal screening reference is evaluated according to Equation (1), and the screening threshold in Equation (2) is defined as follows. The reference used for residual screening is not capped at rated power within this interval.

(1) $$r_{i}=\frac{\left|P_{i}^{\mathrm{SCADA}}-P_{i}^{\mathrm{ref}}\right|}{P_{\mathrm{rated}}}$$

(2) $${\mathrm{flag}}_{i}=1\left[r_{i}>\max\left(3\sigma_{r},0.05\right)\right]$$

Here *σ_r_* denotes the standard deviation of the normalized residual estimated only from the training segment. Points that are subsequently flagged within the training segment are included in this estimate. The threshold is the larger of 3*σ_r_* and 0.05: the first term reflects residual dispersion, whereas the second prevents an excessively narrow criterion and corresponds to a minimum normalized deviation of 77.5 kW for a 1550 kW turbine. A flagged observation does not cause the synchronized SCADA row to be deleted. Instead, only the active-power channel (Patv) is set to not-a-number (NaN), and the screening location is stored separately. The original target-validity mask is retained for evaluation, while screened positions are excluded from the training target loss. Missing values are first forward-filled for up to six steps. Any remaining gaps are then replaced with medians estimated from the training segment, while zero is used only when the corresponding training median is unavailable.

After imputation, physically plausible clipping ranges are applied to selected SCADA channels. Wind speed (Wspd) is restricted to [0, 40] m/s, external temperature (Etmp) to [−50, 60] °C, internal temperature (Itmp) to [−50, 100] °C, blade pitch angles (Pab1–3) to [0, 90]°, and active power (Patv) to [0, 1.05 × 1550] kW. The cleaned records are then converted into the 19 model features. Median–interquartile-range normalization is estimated only from the training segment and is reused for the validation and test segments, which prevents future observations from affecting the normalization statistics.

## 3. Method

### 3.1. Overall Architecture

**Figure 3:**
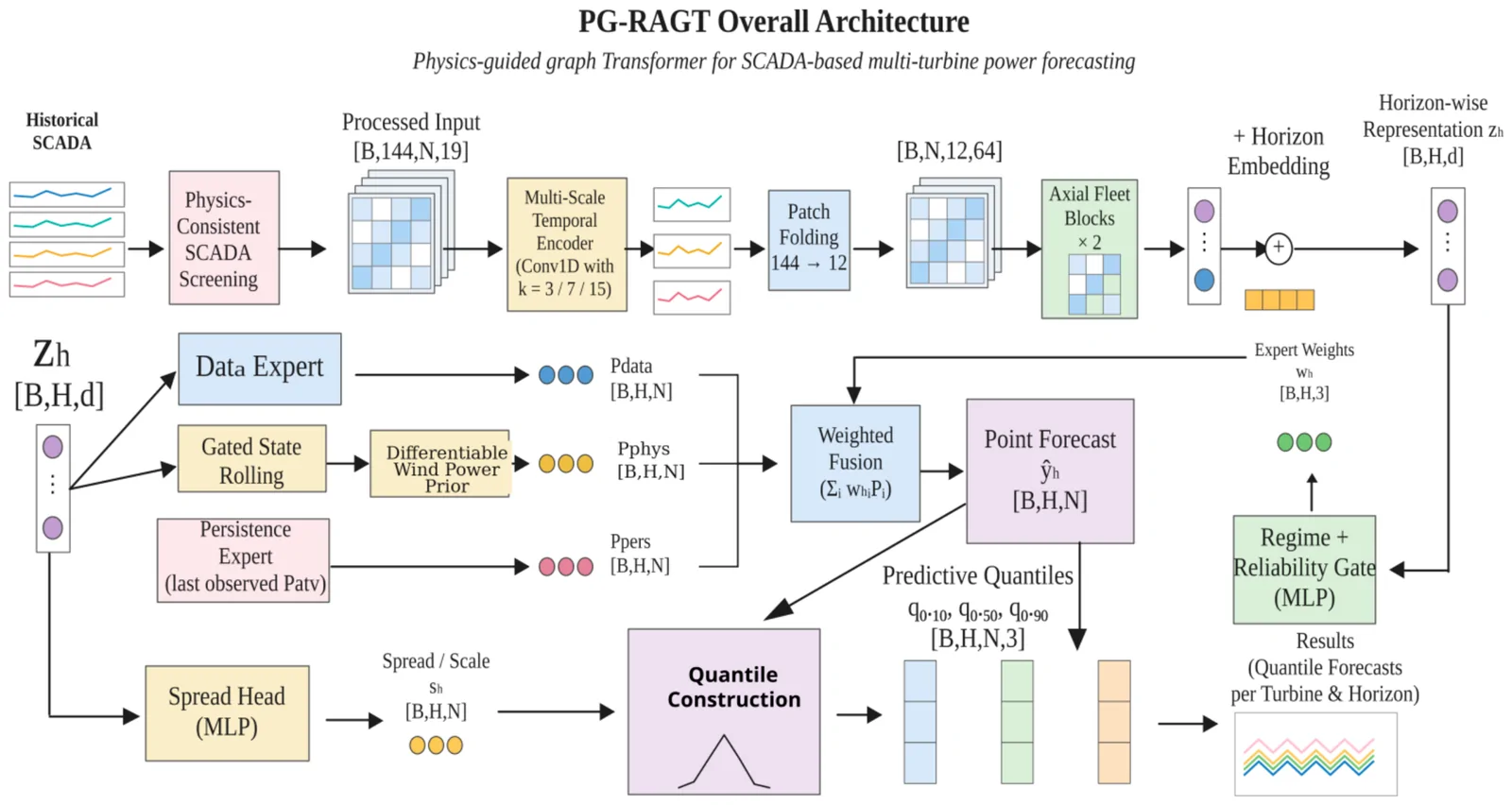
Architecture of the PG-RAGT model. Ellipses denote repetition; colors distinguish modules; arrows show flow.

As shown in Figure 3, PG-RAGT comprises Physics-Consistent SCADA Screening, multi-scale temporal encoding, two Axial Fleet Blocks, Gated Physical-State Rolling, reliability-weighted fusion, and uncertainty estimation. The resulting horizon-wise representation supports point forecasting and auxiliary physical-guidance and uncertainty pathways.

After Physics-Consistent SCADA Screening, the model receives 19 processed SCADA and engineered features from 134 turbines over 144 historical time steps, giving an input tensor of shape [*B*, 144, 134, 19]. Local SCADA variations occur over different short time spans, whereas a single convolution scale provides only one local receptive field. To retain these variations before temporal compression, three parallel one-dimensional convolution branches with kernel sizes of 3, 7, and 15 are applied along the temporal axis. In the implemented stem, each branch produces 32 channels. The GELU-activated branch outputs are concatenated into 96 channels and then fused into a 64-dimensional feature space by a 1 × 1 convolution followed by GELU and dropout. A patch convolution with kernel size and stride 12 subsequently compresses the 144 observations into 12 temporal patches, each corresponding to approximately 2 h, and produces a tensor of shape [*B*, 134, 12, 64]. Learnable positional embeddings and turbine embeddings are added before the representation is passed to two Axial Fleet Blocks.

Temporal dependence and fleet interaction are related, but modeling all turbine-time tokens in one attention operation would mix the two dimensions from the outset. PG-RAGT instead separates them inside each Axial Fleet Block. Temporal attention is first applied independently to the 12 patches of each turbine, after which cross-turbine attention is evaluated among the 134 turbine representations at the same temporal patch. This ordering allows each turbine history to be updated before fleet information is exchanged. From a graph perspective, turbines correspond to nodes, while the cross-turbine attention coefficients act as sample- and patch-dependent interaction weights. Since no fixed adjacency matrix is supplied, an attention-induced fleet interaction representation is formed from the current encoded SCADA state rather than from turbine coordinates or a precomputed correlation matrix.

Once the two Axial Fleet Blocks have been applied, the resulting representation is summarized from two temporal views. The final patch preserves information nearest to the forecasting origin, whereas the mean over all 12 patches provides a broader summary of the preceding 24 h. These vectors are concatenated and projected to obtain the turbine-level context. A separate learnable horizon embedding is subsequently added for each of the six forecast steps, producing the horizon-wise representation *z_h_*. The six forecasts share the same historical context, while the horizon embeddings allow each forecast step to retain horizon-specific information.

The horizon-wise representation *z_h_* supports several forecasting pathways with different assumptions. The data expert maps *z_h_* directly to normalized active power, whereas the persistence expert repeats the last observed active power. In the auxiliary physical pathway, Gated Physical-State Rolling estimates horizon-dependent future states from *z_h_*; those states are then evaluated by the Differentiable Wind Power Prior. A regime-recognition head and a reliability gate assign horizon-specific weights to the three candidates, whose weighted sum gives the final point forecast with shape [*B*, 6, 134]. In parallel, the Spread head predicts two positive offsets used to construct the 0.10 and 0.90 conditional quantiles around the fused point forecast, which serves as the 0.50 quantile. From this organization, Physics-Consistent SCADA Screening mainly concerns training-data consistency, Axial Fleet Blocks provide fleet-level representation, and Gated Physical-State Rolling supplies horizon-dependent states for auxiliary physical guidance.

### 3.2. Physics-Consistent SCADA Screening

Conventional range-based screening can remove measurements outside admissible numerical limits, but a Patv value may remain numerically plausible while departing from a nominal wind speed–power relationship. Physics-Consistent SCADA Screening is therefore applied before feature construction as a targeted training-time quality-control step for the active-power channel rather than as a general sensor-fault detector. In the adopted interval 3 < Wspd < 12.5 m/s, a simplified actuator-disk power relation with *C_p_* = 0.45 provides the nominal screening reference. For each finite Wspd-Patv pair satisfying the screening condition, the absolute difference between observed active power and nominal-reference power is normalized by rated power according to Equation (1). An observation is flagged when this residual exceeds max(3*σ_r_*, 0.05), as defined in Equation (2), where *σ_r_* is computed from training-segment residuals. The first term adapts the criterion to residual dispersion, whereas the lower bound of 0.05 prevents an excessively narrow threshold. Unlike row-wise deletion, only Patv is set to NaN when an observation is flagged; synchronized measurements from the remaining SCADA channels are retained. Before imputation, the screening mask is recorded, while the original target-validity mask is retained for evaluation. Values are forward-filled for up to six steps, and remaining gaps use training-segment medians, with zero used only if a median is unavailable.

To quantify the channel-level effect of the initial range-based screening stage, Table 1 reports the number and proportion of sentinel-value flags for each measurement channel.

Most sentinel-value flags occur in Etmp, whereas relatively few are found in the other listed channels; Prtv denotes the SCADA reactive-power channel. Nominal-reference deviations in active power are concentrated mainly in the upper Partial-Load and Transition regions. As examined quantitatively in Section 4.4.5, the simplified reference does not match the empirical power curve uniformly across the screening interval. A departure from this reference does not by itself indicate sensor failure, because valid pitch-regulated or power-limited operating conditions can produce a similar residual.

### 3.3. Axial Fleet Attention and Attention-Induced Fleet Interaction Modeling

After screening, local temporal patterns must first be represented before fleet interactions are modeled. A single convolution scale would capture local changes through only one receptive field, while direct self-attention over all turbine-time tokens would combine temporal evolution and fleet interaction within the same attention matrix. PG-RAGT handles these two issues in stages. The multi-scale stem uses the three convolution branches described in Section 3.1 and compresses the history into *P* temporal patches. For an input tensor of shape [*B*, *N*, *P*, *D*], the Axial Fleet Block first processes the *P* patch tokens of each turbine by temporal self-attention and then applies cross-turbine self-attention to the *N* turbines at each patch. This factorization follows the general axial-attention principle of applying attention along separate tensor axes [34]. Four attention heads are used with a hidden dimension of *D* = 64. The basic scaled dot-product attention operation is given in Equation (3).

(3) $$\mathrm{Attention}\left(Q,K,V\right)=\mathrm{softmax}\left(\frac{QK^{⊤}}{\sqrt{d_{k}}}\right)V$$

For sample *b*, temporal patch *p*, and attention head *h*, the cross-turbine attention matrix is defined in Equation (4):

(4) $$A_{b,p}^{\left(h\right)}=\mathrm{softmax}\left(\frac{Q_{b,p}^{\left(h\right)}{\left(K_{b,p}^{\left(h\right)}\right)}^{⊤}}{\sqrt{d_{h}}}\right),A_{b,p}^{\left(h\right)}\in {\mathbb{R}}^{N\times N}.$$

For interpretation, the four attention heads are averaged in Equation (5):

(5) $$A_{b,p}=\frac{1}{H}\sum_{h=1}^{H}A_{b,p}^{\left(h\right)}.$$

An element of the head-averaged attention matrix gives the learned mixing weight from source turbine *j* to target turbine *i*. The softmax is applied over source turbines, so each row sums to one.

After temporal attention, the representation is rearranged so that the *N* turbines at the same temporal patch form one attention sequence. Cross-turbine self-attention is then applied independently at each patch, with its mixing coefficients determined by the query and key representations of the current sample. All turbine representations at the same patch participate in standard scaled dot-product self-attention, so the interaction matrix can vary across samples and temporal patches. We interpret this matrix as an attention-induced weighted fleet interaction graph, with turbines as nodes and learned mixing coefficients as directed edge weights. Residual connections, dropout, and LayerNorm are applied around the attention operations, after which a 64-192-64 feed-forward network completes each Axial Fleet Block. The corresponding computational cost is written as follows,

(6) $${\mathrm{Cost}}_{\mathrm{axial}}\approx O\left(BNP^{2}D\right)+O\left(BPN^{2}D\right)$$

where *N* is the number of turbines, *P* is the number of temporal patches, and *D* is the hidden dimension. For the present configuration, *N* = 134, *P* = 12, and *D* = 64, giving an overall complexity of O(*NP*^2^*D* + *PN*^2^*D*). The cross-turbine term accounts for the larger part of the axial-attention cost. The resulting attention matrices provide sample- and patch-dependent fleet interaction weights without a separately supplied adjacency matrix. For reference, GRU [12] and AGCRN [19] have complexities of O(*NLd*^2^) and O(*N*^2^*LF* + *NLd*^2^), respectively; practical efficiency remains dependent on the implementation and hardware configuration. This representation provides the horizon-wise context required by the forecasting heads, but the auxiliary physical branch still faces a different constraint: future Wspd, Etmp, and mean pitch angle are unavailable at forecast time.

**Figure 4:**
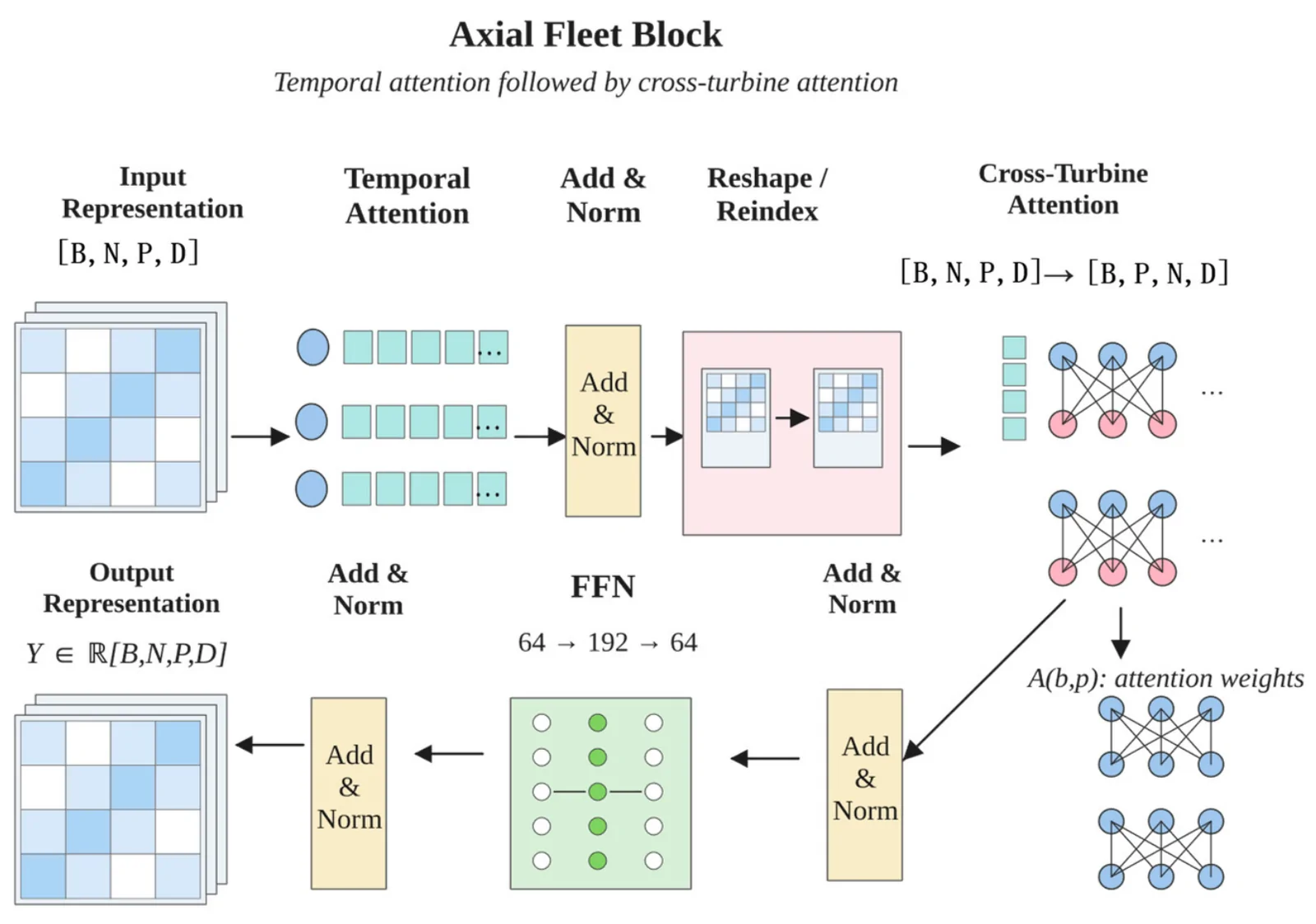
Internal structure of the Axial Fleet Block. Ellipses indicate repeated tokens and turbines; colors distinguish processing stages; arrows show the information flow.

Figure 4 further illustrates that temporal and fleet interactions are processed along different axes rather than being mixed in a single attention operation. Each turbine history is updated first, after which cross-turbine dependencies are estimated from the current patch representations. From this perspective, the interaction weights vary with both the input sample and the temporal patch, while no predefined adjacency matrix is required.

### 3.4. Gated Physical-State Rolling

The Differentiable Wind Power Prior requires a physical state for each forecast horizon, but future SCADA measurements cannot be used without information leakage. Repeating the last observed state would avoid leakage, although the six future states would then be forced to remain unchanged. Gated Physical-State Rolling instead provides forecast-time physical-state closure by allowing bounded, horizon-specific evolution from the same observed origin. For each horizon-wise representation *z_h_*, the State delta + gate module applies a 64-dimensional hidden transformation with GELU and outputs three normalized state increments and three update gates for wind speed, external temperature, and mean pitch angle. The increments are bounded by 0.35 tanh(·), the gates are mapped to (0, 1) by a sigmoid, and the gated increments are cumulatively added to the last observed state *s*_0_. Equations (7) and (8) define this update, after which the states are denormalized and supplied to the Differentiable Wind Power Prior in Equation (9).

(7) $$s_{h}=s_{0}+\sum_{j=1}^{h}\left(g_{j}⊙\Delta s_{j}\right)$$

(8) $$\Delta s_{j}=0.35\tan h\left(W_{\Delta }z_{j}+b_{\Delta }\right),g_{j}=\sigma \left(W_{g}z_{j}+b_{g}\right)$$

(9) $$P_{h}^{\mathrm{aero}}=\frac{0.5\rho \left(T_{h}\right)\left(\pi R^{2}\right)C_{p}\left(\lambda_{\mathrm{TSR}},\beta_{\mathrm{pitch},h}\right)v_{h}^{3}\eta }{1000}$$

Here, Δ*s_j_* denotes the bounded normalized state increment at step *j*, while *g_j_* determines how much of that increment enters the cumulative update. All six states share the observed origin *s*_0_, and the preceding gated increments are retained in later horizons. At the same time, previously predicted future states are not fed back into the State delta + gate module; each increment and gate is generated from its corresponding *z_h_* before cumulative summation. The mechanism is therefore cumulative but non-autoregressive, which separates it from both last-state persistence and recursive state prediction.

The normalized state sequence is converted back to physical units before the aerodynamic prior is evaluated. Wind speed, external temperature, and mean pitch angle are constrained to [0, 40] m/s, [−50, 60] °C, and [0, 90]°, respectively. Air density is computed as *ρ*(*T*) = 1.225 × 288.15/(*T* + 273.15) kg/m^3^. For the SDWPF experiments, the adopted rotor radius is *R* = 38.5 m and the adopted cut-in/rated/cut-out wind speeds are 3.0/12.5/25.0 m/s. Here, “Differentiable Wind Power Prior” denotes the differentiable implementation of a simplified actuator-disk aerodynamic power relation with a semi-empirical power coefficient. The power-coefficient relation follows the semi-empirical *C_p_*(*λ*_TSR_, *β*_pitch_) form reported by Mosayyebi et al. [35]. Its coefficients are given in Equations (10) and (11).

(10) $$\lambda_{i}={\left(\frac{1}{\lambda_{\mathrm{TSR}}+0.08\beta_{\mathrm{pitch}}}-\frac{0.035}{\beta_{\mathrm{pitch}}^{3}+1}\right)}^{-1}$$

(11) $$C_{p}\left(\lambda_{\mathrm{TSR}},\beta_{\mathrm{pitch}}\right)=0.5176\left(\frac{116}{\lambda_{i}}-0.4\beta_{\mathrm{pitch}}-5\right)\exp\left(-\frac{21}{\lambda_{i}}\right)+0.0068\lambda_{\mathrm{TSR}}$$

A fixed *λ*_TSR_ = 7.5 is used as a representative nominal operating-point assumption because rotor-speed information is not included in the three-state physical pathway; a time-varying *λ* = *ωR*/*v* therefore cannot be evaluated at future horizons without introducing an additional predicted rotor-speed state. The power coefficient is clipped to [0, 0.593], while the turbine-specific *C_p_* gain, electromechanical efficiency, and rated-power gain are constrained to [0.80, 1.20], [0.82, 0.97], and [0.95, 1.05], respectively. These gains are trainable model parameters estimated from the training segment only; validation is used for early stopping, and neither validation nor test targets are used to identify them. The separate screening reference uses *C_p_* = 0.45 as a fixed, a priori nominal conversion coefficient rather than the semi-empirical prior; it is not intended to represent the exact turbine-specific power coefficient and was not fitted using validation or test data. In Equation (9), *ρ* has units kg/m^3^, π*R*^2^ has units m^2^, and *v*^3^ has units m^3^/s^3^, whereas *C_p_* and *η* are dimensionless. The numerator is therefore in watts and division by 1000 gives kW. Smooth sigmoid transitions around cut-in, rated, and cut-out wind speeds are used to avoid abrupt regime switching. For turbines with different rotor size, rated speed, or control strategy, these nominal settings require recalibration. Equation (9) reports pre-transition aerodynamic power in kW. Before the physical candidate enters Equation (13), the implementation divides this power by the site-rated power and applies rated-power and operating-region gates. The resulting physical expert output, like the data and persistence expert outputs, is dimensionless in the fusion equation.

### 3.5. Reliability-Weighted Forecast Fusion

The three candidates are based on different assumptions and may perform differently across operating conditions. Fixed fusion weights may therefore be less suitable when the turbine operating state changes. PG-RAGT instead determines the mixture separately for each turbine and forecast horizon through reliability-weighted forecast fusion. The data expert uses a 64-64-1 prediction head with GELU and dropout, and its normalized output is bounded by 1.05 × sigmoid(·) before fusion. By contrast, the persistence expert repeats the latest observed active power, while the auxiliary aerodynamic branch supplies the physical candidate obtained from the rolled states.

Auxiliary regime labels are defined from physical-scale SCADA variables. Shutdown/Low-Output operation is assigned when Wspd < 3 m/s or Patv < 3% of rated power; among the remaining samples, Rated Operation is assigned when Wspd ≥ 12.5 m/s, Patv > 90% of rated power, or the mean pitch angle exceeds 5°, while the others are treated as Partial-Load operation. These future labels are used for supervision but are not supplied during forecasting. Instead, the regime-recognition head maps each 64-dimensional *z_h_* to three logits, and softmax converts them into predicted regime probabilities. The three probabilities are concatenated with *z_h_* to form a 67-dimensional gate input. A 67-32-3 reliability gate with GELU then produces three logits, which are normalized by softmax. In the implementation, the resulting weights correspond to the auxiliary aerodynamic branch, data expert, and persistence expert, respectively. No minimum weight is imposed on any candidate, allowing its contribution to become relatively small when the gate assigns a low fusion weight. The corresponding fusion weights and final point prediction are defined in Equations (12) and (13).

(12) $$w_{h}=\mathrm{softmax}\left(G\left(\left[z_{h},\pi_{h}\right]\right)\right)$$

(13) $${\hat{P}}_{h}=w_{h}^{\mathrm{phys}}P_{h}^{\mathrm{phys}}+w_{h}^{\mathrm{data}}P_{h}^{\mathrm{data}}+w_{h}^{\mathrm{pers}}P_{h}^{\mathrm{pers}}$$

### 3.6. Quantile-Based Prediction Intervals

The fused point prediction describes the expected power output but does not directly quantify predictive uncertainty. Independent quantile prediction does not guarantee the correct ordering of the predicted quantiles. PG-RAGT instead uses the Spread head, implemented as a 64-32-2 network with GELU, to predict two offsets around the fused point forecast. The two raw outputs are transformed by Softplus and increased by 10^−4^, which keeps both spreads positive. The first spread is subtracted from the point prediction to obtain the lower conditional quantile, while the second is added to obtain the upper conditional quantile; the point forecast itself serves as the 0.50 quantile. In this way, *q*_0.10_ ≤ *q*_0.50_ ≤ *q*_0.90_ is preserved by construction rather than corrected after prediction. All three outputs are clipped to the normalized range [0, 1.10]. For the nominal 80% prediction interval evaluated in this study, *q*_0.10_(*x*) and *q*_0.90_(*x*) are used as the lower and upper bounds, respectively, as summarized in Equation (14). The point, interval, state, and regime outputs are trained jointly, which leads to the objective described next.

(14) $${\mathrm{PI}}_{80}\left(x\right)=\left[q_{0.10}\left(x\right),q_{0.90}\left(x\right)\right]$$

### 3.7. Training Objective

PG-RAGT is trained using a joint objective combining point forecasting, quantile estimation, future-state supervision, operating-regime classification, ramp consistency, and physics-guided regularization, as follows:

(15) $$L=L_{\mathrm{point}}+0.25L_{\mathrm{quantile}}+0.15L_{\mathrm{state}}+0.10L_{\mathrm{regime}}+0.10L_{\mathrm{ramp}}+0.03L_{\mathrm{physics}}$$

The point term uses a masked Smooth L1 loss with Transition parameter *δ* = 0.05 between the fused prediction and the observed power. Conditional quantiles are supervised by the pinball loss, while future wind speed, external temperature, and mean pitch angle use a masked Smooth L1 loss with *δ* = 0.10. A three-class cross-entropy term supervises the regime-recognition head. The ramp term measures the absolute difference between predicted and observed power increments across neighboring horizons. Physics consistency is imposed only on Partial-Load samples: a masked Smooth L1 loss with *δ* = 0.05 regularizes the data expert toward the detached output of the auxiliary aerodynamic branch. Because that reference is detached, the gradient adjusts the data-driven branch without forcing the Differentiable Wind Power Prior to follow the data expert. As written directly in Equation (15), the weights are 1.0 for the point term and 0.25, 0.15, 0.10, 0.10, and 0.03 for the quantile, state, regime, ramp, and physics terms, respectively. Point forecasting remains the primary optimization target, while the other losses provide auxiliary supervision.

(16) $$L_{\mathrm{quantile}}=\frac{1}{N\left|T\right|}\sum_{i=1}^{N}\sum_{\tau \in T}\rho_{\tau }\left(y_{i}-{\hat{q}}_{i,\tau }\right),T=\left\{0.10,0.50,0.90\right\}$$

(17) $$\rho_{\tau }\left(u\right)=\max\left(\tau u,\left(\tau -1\right)u\right)$$

## 4. Experiments

After the common experimental setup, the evaluation is organized into three parts: component-level validation of PG-RAGT, comparison with existing forecasting methods, and application-oriented analysis across forecast horizons, operating conditions, turbines, prediction intervals, screening behavior, and an external wind farm.

### 4.1. Experimental Setup

#### 4.1.1. Baseline Models and Implementation Settings

Table 2 compares the baseline models in terms of trainable parameter counts, model-fitting times, and comparison roles.

Seven deterministic baselines are considered: persistence, GRU [12], PatchTST [15], AGCRN [19], aerodynamic prior, STGCformer [22], and STV-PGNN [24]. Persistence provides a last-value reference, GRU represents recurrent temporal modeling, and PatchTST provides a patch-based Transformer baseline without explicit fleet interaction. AGCRN is the primary adaptive graph baseline, whereas aerodynamic prior repeats the last observed physical state before applying the same simplified Differentiable Wind Power Prior. STGCformer and STV-PGNN provide recent graph Transformer and physics-guided spatiotemporal graph references. All deterministic models use the same chronological split, 144-step historical window, 6-step forecast horizon, and evaluation protocol. The main comparison uses the common preprocessing pipeline, including Physics-Consistent SCADA Screening; its influence on AGCRN is checked separately below.

PG-RAGT uses a hidden dimension of 64, four attention heads, two Axial Fleet Blocks, a 192-dimensional feed-forward network, dropout of 0.10, and a patch length of 12. GRU and AGCRN use hidden dimensions of 152 and 174 for the parameter-matched comparison, giving 219,342 and 220,464 trainable parameters; PatchTST contains 210,342 parameters, while STGCformer and STV-PGNN contain 225,783 and 228,816. The 402 trainable parameters of aerodynamic prior are the three turbine-specific calibration parameters for each of 134 turbines. Hyperparameters were fixed before test evaluation, and no test-set-based tuning was performed. The common optimizer and training protocol were retained across the trainable baselines, with architecture-specific dimensions used as reported in Table 2. The implemented AGCRN uses an adaptive adjacency shared across input samples, whereas PG-RAGT recomputes cross-turbine mixing from the current patch representations.

To isolate the effect of preprocessing from model architecture, we additionally performed a controlled AGCRN sensitivity run using conventional preprocessing, with Physics-Consistent SCADA Screening disabled while retaining the same parameter-matched AGCRN configuration and training settings. For seed 2026, AGCRN changed from RMSE = 109.87 kW and MAE = 63.96 kW with screening to RMSE = 110.13 kW and MAE = 63.61 kW without screening. Because the changes are small and opposite in direction for RMSE and MAE, they are treated as a preprocessing-sensitivity check rather than evidence that screening generally improves AGCRN. Under the same conventional preprocessing, the existing PG-RAGT no-screening ablation still gives lower RMSE and MAE (108.66 and 58.89 kW) than AGCRN (110.13 and 63.61 kW), indicating that the architecture-level difference is not explained solely by the screening step.

A reduced-model check was also performed without retuning the remaining architecture. Replacing the two Axial Fleet Blocks with one block reduces trainable parameters from 219,809 to 161,313 (−26.6%). On seed 2026, RMSE changes from 106.69 to 107.17 kW, an increase of about 0.45%, while MAE changes from 57.12 to 58.75 kW. Under the same latency protocol using an NVIDIA GeForce RTX 4050 Laptop GPU (NVIDIA Corporation, Santa Clara, CA, USA), mean forward-pass latency falls from 0.868 to 0.715 ms per forecasting window (−17.7%). Thus, the two-block model retains the best overall accuracy, while the one-block variant provides a comparatively favorable accuracy–efficiency trade-off for resource-constrained use.

#### 4.1.2. Evaluation Metrics and Statistical Testing

Deterministic performance is evaluated using mean absolute error (MAE), root mean square error (RMSE), coefficient of determination (*R*^2^), and mean absolute percentage error (MAPE) on samples marked as valid by the target-validity mask. MAPE is computed only for samples with power output exceeding 5% of the rated value (77.5 kW). Higher *R*^2^ indicates a better fit, whereas lower MAE, RMSE, and MAPE indicate higher forecasting accuracy.

(18) $$\mathrm{MAE}=\frac{1}{n}\sum_{i=1}^{n}\left|y_{i}-{\hat{y}}_{i}\right|$$

(19) $$\mathrm{RMSE}=\sqrt{\frac{1}{n}\sum_{i=1}^{n}{\left(y_{i}-{\hat{y}}_{i}\right)}^{2}}$$

(20) $$R^{2}=1-\frac{\sum_{i=1}^{n}{\left(y_{i}-{\hat{y}}_{i}\right)}^{2}}{\sum_{i=1}^{n}{\left(y_{i}-\bar{y}\right)}^{2}}$$

(21) $$\mathrm{MAPE}=\frac{100\%}{\left|S\right|}\sum_{i\in S}\left|\frac{y_{i}-{\hat{y}}_{i}}{y_{i}}\right|,S=\left\{i:y_{i}>0.05P_{\mathrm{rated}}\right\}$$

Prediction uncertainty is evaluated using prediction interval coverage probability (PICP), mean prediction interval width (MPIW), and prediction interval normalized average width (PINAW). PICP measures empirical coverage, MPIW measures interval width, and PINAW divides MPIW by the site-specific adopted power reference *P*_rated_. Accordingly, *P*_rated_ = 1550 kW is used for SDWPF and 2000 kW for Penmanshiel. For the probabilistic baseline comparison, mean pinball loss and the 80% interval score are also reported, following the loss-oriented scoring rules of Gneiting and Raftery [36].

(22) $$\mathrm{PICP}=\frac{1}{n}\sum_{i=1}^{n}c_{i}$$

(23) $$\mathrm{MPIW}=\frac{1}{n}\sum_{i=1}^{n}(U_{i}-L_{i})$$

(24) $$\mathrm{PINAW}=\frac{\mathrm{MPIW}}{P_{\mathrm{rated}}}$$

(25) $$\mathrm{MPL}=\frac{1}{3n}\sum_{i=1}^{n}\sum_{\tau \in Q}\max\left[\tau e_{i,\tau },\left(\tau -1\right)e_{i,\tau }\right]$$

(26) $${\mathrm{IS}}_{\alpha }=\frac{1}{n}\sum_{i=1}^{n}\left[\left(U_{i}-L_{i}\right)+\frac{2}{\alpha }\max\left(L_{i}-y_{i},0\right)+\frac{2}{\alpha }\max\left(y_{i}-U_{i},0\right)\right]$$

Here, $e_{i,\tau }=y_{i}-{\hat{q}}_{i,\tau }$ denotes the quantile error; $Q=\left\{0.10,\;0.50,\;0.90\right\}$; $L_{i}={\hat{q}}_{i,0.10}$; and $U_{i}={\hat{q}}_{i,0.90}$. All values are evaluated in kW. For the nominal 80% prediction interval, α=0.20 and 2/α=10. Both scores are averaged over the same valid test targets; lower values are better.

The contributions of PG-RAGT are further evaluated through ablation experiments and Diebold–Mariano testing [37]. The statistical significance of the ablation differences is assessed using the Diebold–Mariano test based on the per-window MAE across 5287 test windows. The long-run variance of the test statistic is estimated using the Newey–West estimator to account for serial dependence among adjacent test windows. Moving-block bootstrap 95% confidence intervals (CIs) are computed using a block length of 144 windows, 5000 resamples, and a random seed of 2026; paired Wilcoxon signed-rank tests are conducted using a two-sided alternative.

(27) $$d_{i}={\mathrm{MAE}}_{i}^{\left(1\right)}-{\mathrm{MAE}}_{i}^{\left(2\right)},\bar{d}=\frac{1}{N}\sum_{i=1}^{N}d_{i}$$

(28) $$\mathrm{DM}=\frac{\bar{d}}{\sqrt{\hat{\Omega }/N}}$$

Here, $\hat{\Omega }$ denotes the long-run variance of the loss differential estimated using the Newey–West estimator. For reporting, MAE degradation is defined as ablation minus full model, whereas the DM statistic retains the full-minus-ablation loss-difference convention used in the test.

#### 4.1.3. Reproducibility Controls

The data pipeline is based on deterministic operations, including sentinel-value screening, Physics-Consistent SCADA Screening, causal missing-value imputation, feature engineering, median–interquartile-range (IQR) normalization, and temporal segmentation. Forward filling is limited to six past steps, while remaining gaps use training-segment medians; preprocessing thresholds and normalization statistics are not estimated from validation or test observations. The software environment used Python 3.11.9, PyTorch 2.6.0+cu124 (CUDA 12.4 runtime), NumPy 2.4.6, pandas 3.0.3, and SciPy 1.17.1.

(29) $$\tilde{x}=\frac{x-\mathrm{median}\left(x\right)}{Q_{3}-Q_{1}}$$

Normalization statistics are estimated exclusively from the training set and stored with each model checkpoint. For the SDWPF trainable neural models, AdamW is used with a target learning rate of 3 × 10^−4^ and a weight decay of 10^−4^. The maximum training budget is 40 epochs, while cosine annealing is applied after two warm-up epochs. A batch size of 16 is used, gradient accumulation is set to one step, and the gradient norm is clipped at 1.0. On CUDA devices, automatic mixed precision is enabled during both training and inference, with gradient scaling applied before each optimizer update.

The sliding-window configuration differs slightly between model fitting and evaluation. Training windows use a stride of two SCADA steps to reduce redundancy, whereas validation and test windows use a stride of one step for denser evaluation. Training samples are shuffled with a seeded PyTorch generator, while validation and test samples retain their generated order. Early stopping is based on validation RMSE with a patience of five epochs. The main seed 2026 PG-RAGT run reaches its best validation epoch at epoch 11 and stops after 16 epochs. All reported multi-seed comparisons use the same CUDA/automatic-mixed-precision setting on an NVIDIA GeForce RTX 4050 Laptop GPU; deterministic CUDA execution is not forced, so the three-seed analysis is interpreted as a robustness check across independently seeded training runs rather than as a complete decomposition of initialization and numerical-runtime variability.

Table 2 reports approximate model-fitting times on the laptop GPU. For the audited PG-RAGT run, the broader wall-clock interval including preprocessing is about 17.4 min, while the table retains the previously reported model-fitting time of about 11.6 min. During inference, a prepared batch of 16 full-farm windows requires 13.9 ms on average, corresponding to approximately 0.87 ms per window; disk I/O, preprocessing, and host-to-device transfer are excluded from this forward-pass measurement. Each window contains the 134-turbine history and produces all six forecast horizons.

### 4.2. Component-Level Validation of PG-RAGT

Before comparing PG-RAGT with other forecasting models, the internal behavior of its auxiliary pathways is examined first. Table 3, Table 4 and Table 5 provide three complementary diagnostics: Table 3 evaluates the horizon-dependent physical states generated by Gated Physical-State Rolling, Table 4 reports the corresponding state-update gates under different operating regimes, and Table 5 compares the individual loss components between the first and final training epochs. These analyses examine whether the rolled states change with forecast horizon, whether the three physical variables receive different update strengths, and whether the auxiliary objectives remain active during optimization. Table 6 and Table 7 then assess whether Gated Physical-State Rolling, Physics-Consistent SCADA Screening, and cross-turbine attention produce measurable changes in forecasting performance.

The horizon-dependent states produced by Gated Physical-State Rolling are first evaluated through the mean absolute errors of wind speed, external temperature, and mean pitch angle at each forecast step. The results are reported in Table 3.

As shown in Table 3, estimation errors increase gradually as the forecast horizon moves farther from the observed state. Wind speed MAE rises from 0.303 m/s at 10 min to 0.720 m/s at 60 min, external-temperature MAE increases from 0.199 to 0.816 °C, and mean-pitch-angle MAE increases from 2.622° to 9.463°. A similar horizon-dependent pattern is therefore observed for all three variables. Because the six future states are generated from horizon-specific representations without autoregressive feedback, this increase should not be interpreted as error propagation from one predicted state to the next. Rather, conditions farther from the observed origin are comparatively more difficult to infer from the available SCADA history. The results indicate that Gated Physical-State Rolling produces changing future states instead of simply repeating the last observation, although the estimation error becomes larger at longer horizons.

The state-update gates provide a second view of how the rolling mechanism uses the three physical variables under different operating conditions. Their distributions are summarized in Table 4.

Table 4 shows a clear difference among the three state-update pathways. The overall mean wind speed gate is 0.157, compared with 0.142 for external temperature and 0.047 for mean pitch angle. Under Partial-Load operation, the wind speed and external-temperature gates increase to 0.191 and 0.189, respectively, while the mean-pitch-angle gate remains lower at 0.031. Because these gates scale bounded normalized increments, their numerical values do not directly represent changes in m/s, °C, or degrees. From the model behavior reflected in the table, wind speed and external-temperature evolution receive comparatively larger updates, whereas the mean-pitch-angle pathway remains more conservative.

The activity of the auxiliary objectives is examined next by comparing each loss component at the first and final epochs, as reported in Table 5.

The changes in Table 5 indicate that most auxiliary objectives remain active during training, although their magnitudes differ. From the first to the final epoch, *L*_point_, *L*_quantile_, *L*_state_, *L*_regime_, and *L*_physics_ decrease by approximately 22–57%, whereas *L*_ramp_ changes by only about 1%. The reduction is largest for the physics-consistency term, while the ramp term remains almost unchanged. It is worth noting that improvements observed later under ramp conditions therefore cannot be attributed to *L*_ramp_ alone, because this term constrains adjacent horizon-to-horizon power changes rather than the calm/ramp window classification used in Section 4.4.2.

The learned reliability gate is dominated by the persistence and data experts rather than by the direct aerodynamic candidate. Averaged over the SDWPF test set, the mean weights are 0.29% for the physical candidate, 36.01% for the data expert, and 63.70% for persistence. The physical weight remains small in Shutdown/Low Output (0.26%), Partial Load (0.30%), and Rated Operation (0.55%). A clearer pattern appears across forecast horizons: persistence decreases from 86.68% at 10 min to 46.34% at 60 min, whereas the data expert increases from 13.26% to 53.11%; the physical weight rises only from 0.06% to 0.54%. The physical pathway therefore acts mainly through future-state supervision and the physics-consistency regularizer, while its direct contribution to the final mixture is small.

These internal diagnostics describe how the auxiliary pathways behave, but they do not directly quantify the contribution of each task-specific design to the final forecast. To address this question, the three components are ablated separately. The rolling ablation repeats the last observed physical state across all six horizons. The corresponding deterministic results are reported in Table 6.

Table 6 separates the stable and training-run-dependent component effects. Removing cross-turbine attention gives the largest and most consistent degradation, with a paired RMSE increase of 8.80 ± 2.25 kW across the three seeds. Physics-Consistent SCADA Screening has a smaller mean effect of 2.06 ± 2.25 kW, including one near-zero RMSE reversal (−0.14 kW at seed 2027). Gated Physical-State Rolling yields a paired RMSE change of 0.80 ± 1.27 kW and a paired MAE change of 1.13 ± 0.53 kW; one seed shows a small RMSE reversal (−0.52 kW), while MAE degrades in all three runs when rolling is removed. The cross-turbine pathway is therefore the clearest accuracy contributor, whereas screening and rolling provide smaller, more training-run-sensitive effects. The role of Gated Physical-State Rolling is consequently interpreted primarily as enabling horizon-dependent physical guidance without future SCADA, not as the dominant source of deterministic RMSE improvement.

Table 7 reports the paired window-level statistical tests for the main seed 2026 and should be read together with the cross-seed variability in Table 6. For this seed, removing cross-turbine attention, Physics-Consistent SCADA Screening, and Gated Physical-State Rolling gives Diebold–Mariano statistics of −9.20, −6.86, and −3.84, respectively. The corresponding moving-block bootstrap 95% confidence intervals for MAE degradation are [3.86, 6.83], [1.16, 2.38], and [0.62, 2.79] kW. These tests establish paired differences within the main run, whereas the three-seed results show that the smaller screening and rolling effects are less stable across training runs.

### 4.3. Comparison with Existing Forecasting Methods

#### Overall Forecasting Performance

**Figure 5:**
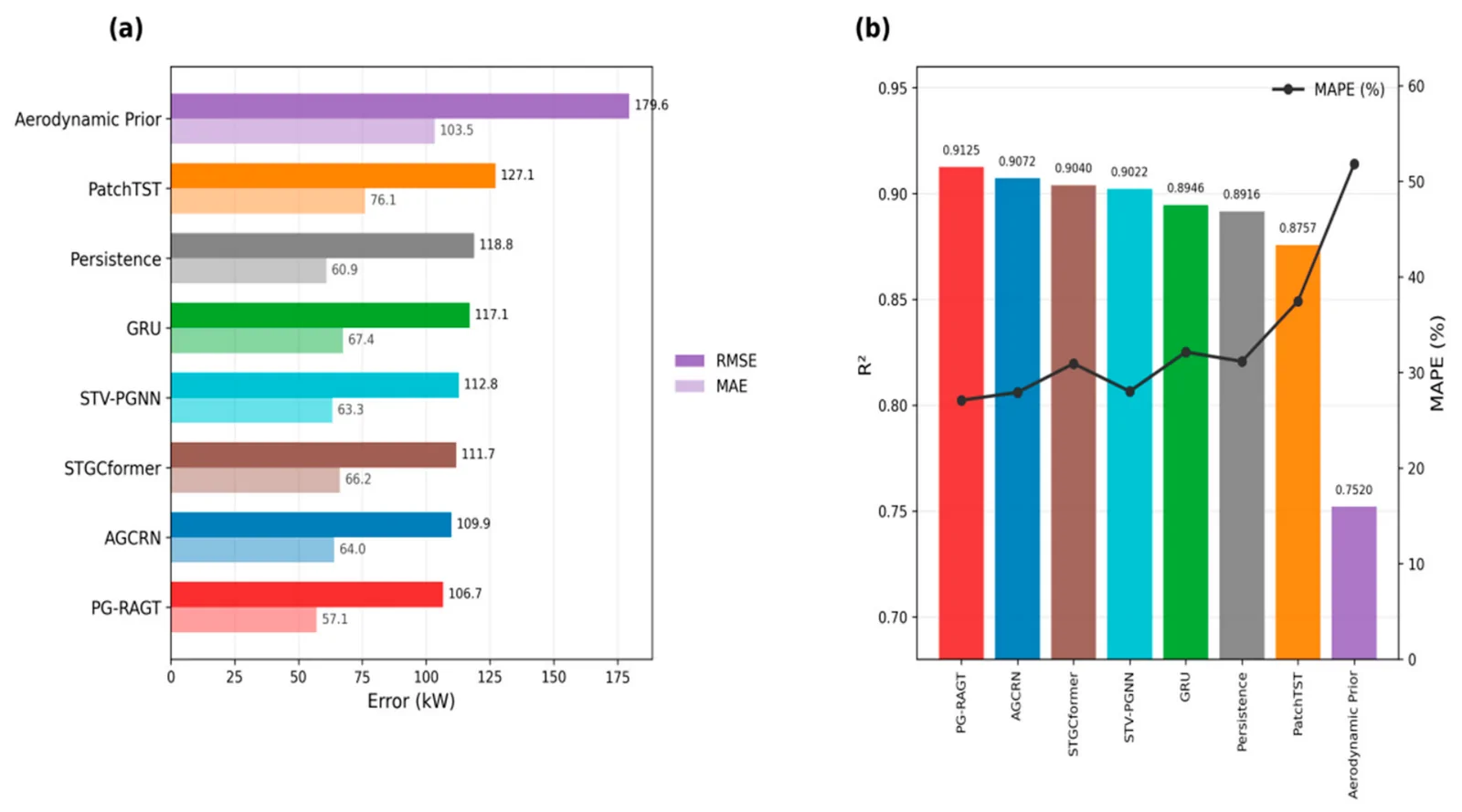
Deterministic forecasting performance across all evaluated models: (**a**) RMSE and MAE; (**b**) *R*^2^ and MAPE. In (**a**), darker and lighter bars indicate RMSE and MAE, respectively, with colors distinguishing models. In (**b**), colored bars show R^2^ and the black line shows MAPE.

To evaluate the overall forecasting performance of PG-RAGT, comparisons are conducted with persistence, GRU, PatchTST, AGCRN, aerodynamic prior, STGCformer, and STV-PGNN under the same chronological split and evaluation protocol. These baselines cover last-value and physics-based references, recurrent and patch-based temporal models, adaptive graph modeling, and two recent graph-based methods. The numerical results are reported in Table 8, while Figure 5 provides the corresponding visual comparison of the main accuracy metrics.

As shown in Table 8 and Figure 5, PG-RAGT achieves *R*^2^ = 0.9125, RMSE = 106.69 kW, MAE = 57.12 kW, and MAPE = 27.09%, giving the best overall deterministic performance among the evaluated models. Among the two recent baselines, STGCformer records an RMSE of 111.74 kW and STV-PGNN records 112.75 kW, so both remain above PG-RAGT under the common protocol. Persistence remains competitive because of strong short-term power inertia, whereas aerodynamic prior gives the largest errors under its persistence-style physical-state assumption. GRU and PatchTST provide temporal references, while AGCRN further reduces error through adaptive graph modeling. From the overall comparison, temporal modeling alone does not fully account for the observed performance, and fleet interaction provides an additional source of predictive information.

The comparison with GRU helps separate the observed gain from model size. PG-RAGT and the parameter-matched GRU contain 219,809 and 219,342 trainable parameters, respectively, yet PG-RAGT reduces RMSE by about 8.9% and MAE by about 15.3%. With nearly matched capacity, the lower errors are more consistent with differences in model structure, including fleet interaction and physics-guided pathways, than with a simple increase in parameter count.

A closer comparison with AGCRN shows a more competitive pattern. PG-RAGT reduces RMSE by about 2.9% and MAE by about 10.7%, so the improvement is more pronounced for typical absolute errors than for errors receiving greater weight under the squared-error metric. The aggregate metrics do not identify how the extreme-error tail changes, but they do show that the gain is not uniform across error measures. Structurally, AGCRN uses an adaptive adjacency shared across input samples, whereas PG-RAGT recomputes cross-turbine attention weights from the current patch representations. The structural difference is therefore in the weighting mechanism. Table 6 further shows that removing the cross-turbine interaction pathway increases forecasting error.

Against persistence, PG-RAGT gives an RMSE skill score of 10.15% and an MAE skill score of 6.21%, using $\mathrm{Skill}\;=\;1\;-\;\frac{E_{\mathrm{model}}}{E_{\mathrm{Persistence}}}$. RMSE skill remains positive at every forecast horizon and ranges from about 7.14% to 10.79%. These values provide a scale-independent practical comparison with the last-value forecast. For a non-overlapping, fixed 60 min ahead evaluation, we retained only forecast step 6 and counted each unique target time once. Using the same 706,952 valid turbine–time pairs (134 turbines, 5281 contributing timestamps), the accumulated absolute energy forecast error, computed as $\sum \left|P_{\mathrm{pred}}\;-\;P_{\mathrm{obs}}\right|\;\times \;\frac{10}{60}\;/\;1000$, is 9173.06 MWh for PG-RAGT and 9726.93 MWh for persistence, a 553.87 MWh (5.69%) reduction over this evaluated test period. This measures a reduction in absolute prediction error, not additional generated energy or monetary savings; summing all six overlapping forecast horizons would not be a valid operational total.

**Figure 6:**
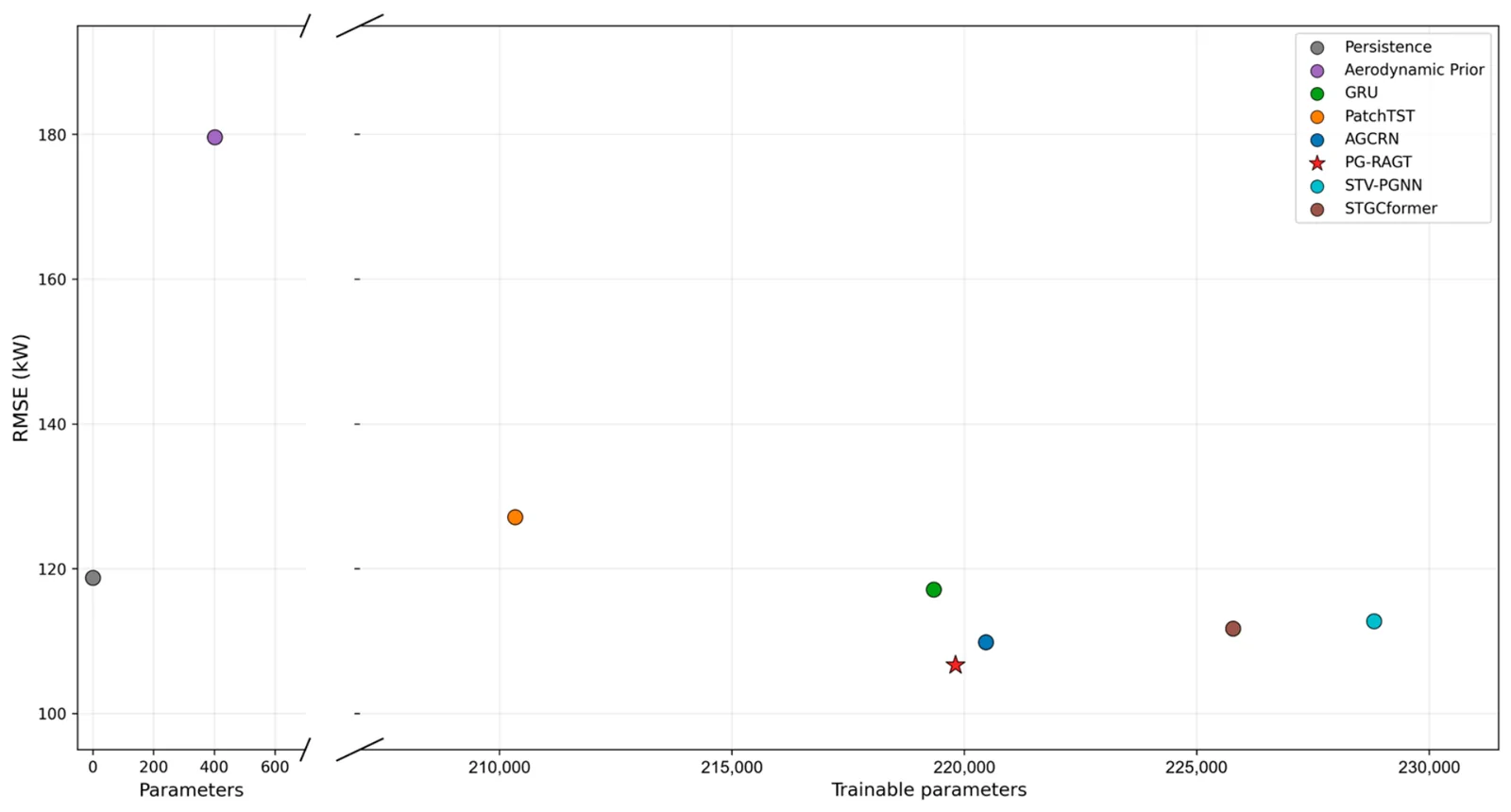
Accuracy–capacity trade-off among the evaluated models. The star marks PG-RAGT.

Figure 6 compares forecasting accuracy with model capacity to examine whether the performance gain of PG-RAGT can be explained mainly by a larger parameter count.

As shown in Figure 6, PG-RAGT and the five trainable neural baselines are located in a relatively similar parameter range of approximately 2.1–2.3 × 10^5^ trainable parameters, while PG-RAGT retains the lowest RMSE. The result makes model capacity alone a less sufficient explanation for the observed accuracy difference. Table 9 therefore provides a further check of whether the ranking remains stable under different random initializations.

Training-run sensitivity is assessed through three independently seeded runs for each trainable neural model, with the corresponding results summarized in Table 9.

Across seeds 2025–2027, PG-RAGT retains the lowest mean RMSE (107.19 ± 1.17 kW) and MAE (57.42 ± 0.50 kW), with mean *R*^2^ = 0.9116 ± 0.0020. The corresponding mean RMSE values are 109.70 ± 0.54 kW for AGCRN, 112.04 ± 0.43 kW for STGCformer, 112.92 ± 0.32 kW for STV-PGNN, 117.92 ± 1.64 kW for GRU, and 130.64 ± 3.54 kW for PatchTST. The ranking is comparatively stable across these three runs, although three seeds do not provide a complete characterization of training variability. The result is therefore used as a robustness check rather than as a claim of deterministic reproducibility.

### 4.4. Application-Oriented Evaluation

The preceding experiments establish the aggregate accuracy and component-level behavior of PG-RAGT. The application-oriented analysis then considers whether the relative performance changes with forecast horizon, operating condition, turbine, uncertainty output, screening behavior, or wind farm setting. The final external-site experiment is validation after retraining rather than direct transfer.

#### 4.4.1. Multi-Horizon Forecasting Performance

**Figure 7:**
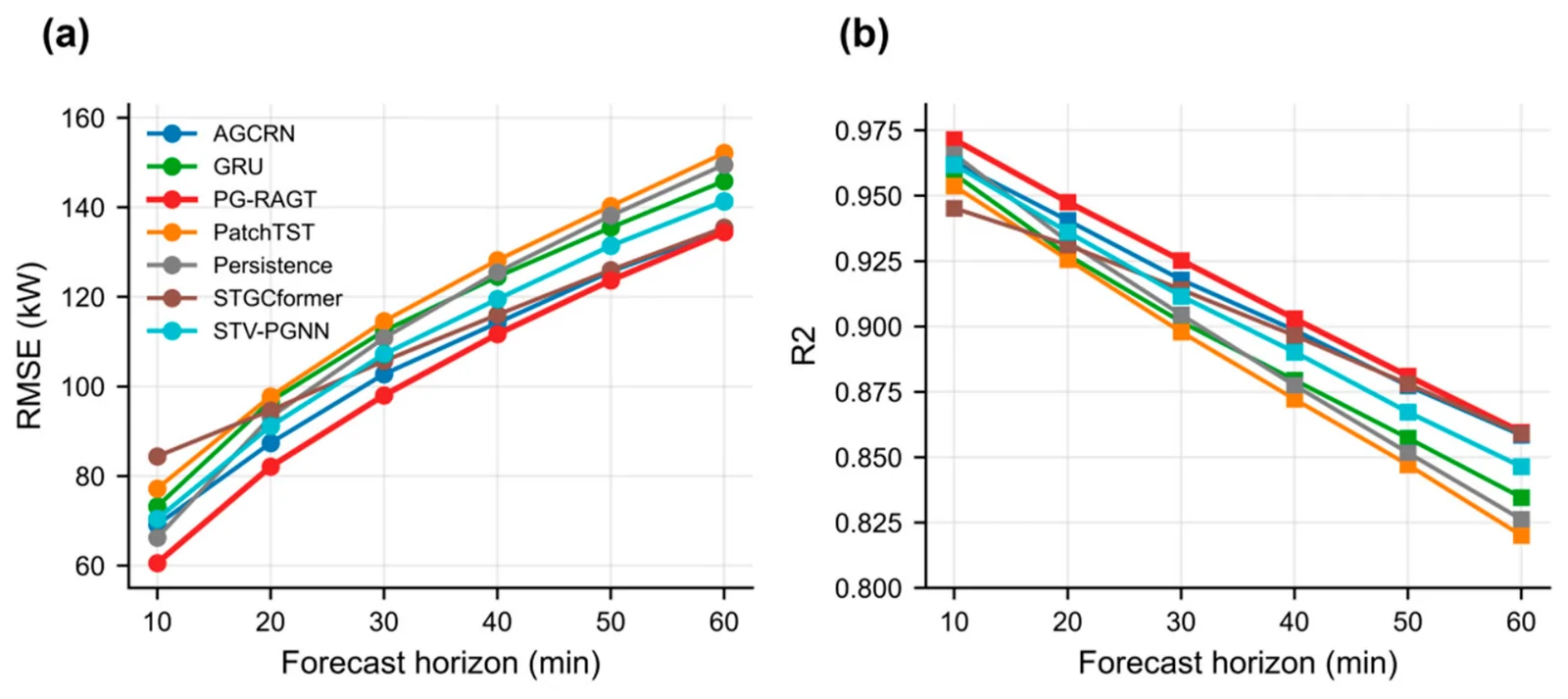
Multi-horizon forecasting performance across the evaluated models: (**a**) RMSE and (**b**) *R*^2^.

Figure 7 compares RMSE and *R*^2^ over the six forecast horizons to examine how the relative advantage changes as the prediction target moves farther from the observed history.

As shown in Figure 7, PG-RAGT maintains the lowest RMSE among the evaluated trainable models at all six forecast horizons, although its margin over AGCRN gradually narrows as lead time increases. The corresponding *R*^2^ curves show the same general tendency as the prediction target moves farther from the observed history. Because all six point forecasts are generated directly from the shared historical context and separate horizon embeddings, this pattern is not caused by autoregressive error propagation. A more plausible explanation is that the available SCADA history becomes less informative for farther targets, while the rolled physical states are also estimated farther from their common observed origin. Persistence shows the complementary behavior, remaining more competitive at short lead times and degrading more clearly as the horizon increases. The corresponding per-horizon RMSE and MAE values for PG-RAGT and AGCRN, together with the relative reductions, are summarized in Table 10.

#### 4.4.2. Ramp-Event and Operating-Regime Performance

**Figure 8:**
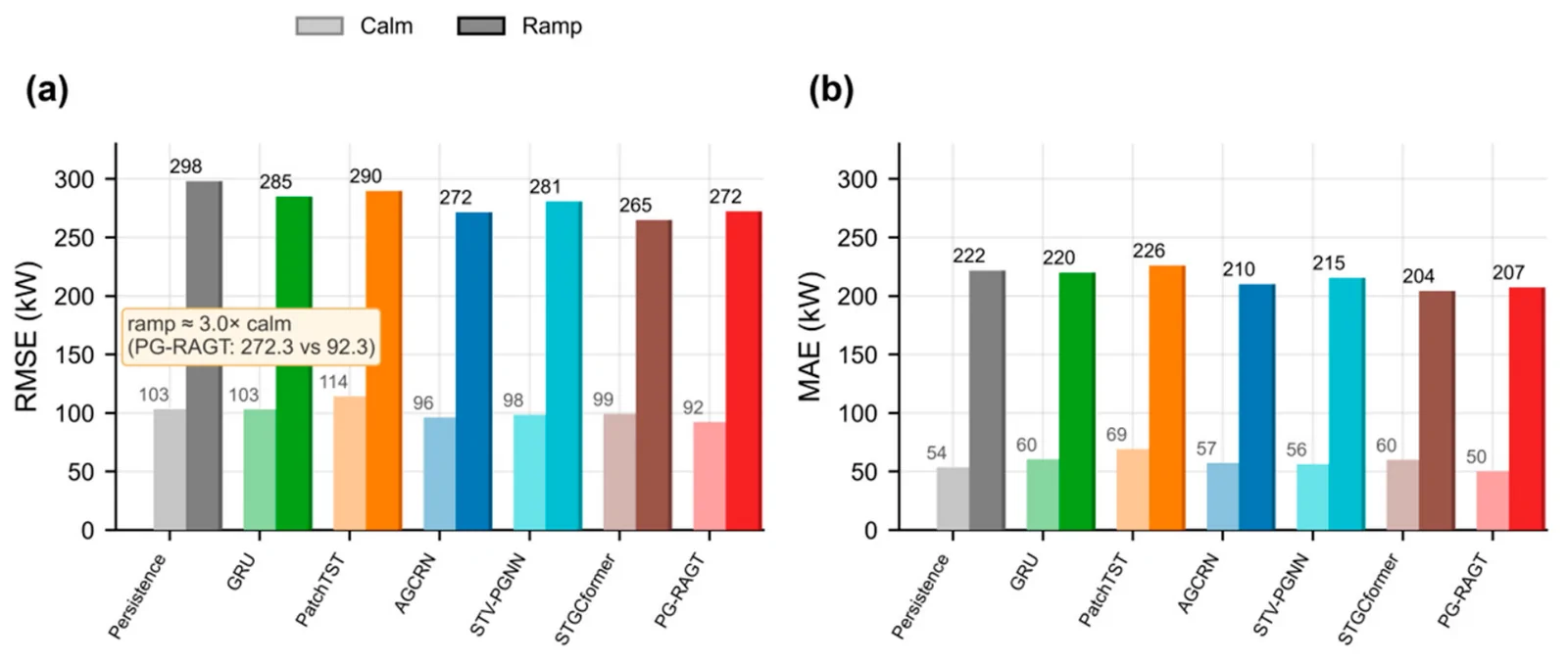
Forecasting errors under calm and ramp conditions: (**a**) RMSE and (**b**) MAE. Colors distinguish models, while lighter and darker bars indicate calm and ramp conditions, respectively.

To examine whether the relative performance changes when power varies rapidly, Figure 8 compares forecasting errors under calm and ramp conditions; the ramp criterion is defined below using the same chronological test windows.

Ramp conditions are identified on the same 5287 test windows. A window is classified as a ramp event when at least 20% of the turbines exhibit a 10 min power change exceeding 20% of the rated power (310 kW) within the forecast horizon, as defined in Equations (30) and (31).

(30) $$R_{w}=\left\{k:\underset{h}{\max}\left|P_{k,t+h+1}-P_{k,t+h}\right|>0.2P_{\mathrm{rated}}\right\}$$

(31) $${\mathrm{Ramp}}_{w}\Leftrightarrow \frac{\left|R_{w}\right|}{N_{\mathrm{turb}}}\geq 0.2,N_{\mathrm{turb}}=134$$

This criterion partitions the same 5287 chronological test windows into calm and ramp-event subsets for the following comparison.

The resulting calm/ramp errors are summarized in Table 11.

Figure 8 and Table 11 show that ramp events substantially increase forecasting difficulty for every evaluated model, with ramp RMSE reaching roughly 2.5–3.0 times the corresponding calm-condition value. Under calm conditions, PG-RAGT gives the lowest RMSE (92.26 kW) and MAE (50.25 kW). During ramps, however, STGCformer gives the lowest RMSE (264.95 kW) and MAE (204.27 kW), whereas PG-RAGT records 272.28 kW and 207.34 kW, respectively. From this comparison, the advantage of PG-RAGT is more evident under calm conditions than during rapid power changes. More generally, the large calm-to-ramp increase indicates that abrupt power variation remains a comparatively difficult forecasting condition despite the auxiliary change-matching loss used during training.

Ramp behavior provides one view of forecasting difficulty, while operating regime provides a complementary view of performance across turbine states. The test samples are therefore grouped into Shutdown/Low Output, Partial Load, and Rated Operation using the same Wspd, Patv, and mean-pitch-angle criteria used for regime supervision, and the corresponding RMSE and *R*^2^ values are reported in Table 12.

Table 12 shows that the relative performance changes with operating regime. In Partial Load, PG-RAGT obtains the lowest RMSE (140.82 kW) and the highest *R*^2^ (0.793); STGCformer and STV-PGNN record 144.83 and 146.89 kW, respectively. Shutdown/Low Output gives a different ordering, with aerodynamic prior at 40.06 kW and PG-RAGT at 44.33 kW; the negative *R*^2^ values in this low-variance regime should be interpreted cautiously. Under Rated Operation, persistence remains best at 199.07 kW, while STGCformer (200.90 kW) and PG-RAGT (202.25 kW) are close. The benefit of PG-RAGT is therefore most visible in Partial Load rather than uniform across all turbine states. From actual operating behavior, saturation and low-output regimes contain less model-relevant variation, while Partial Load retains a steeper wind speed–power relationship and stronger scope for fleet-state information to reduce error.

#### 4.4.3. Fleet-Level Performance

**Figure 9:**
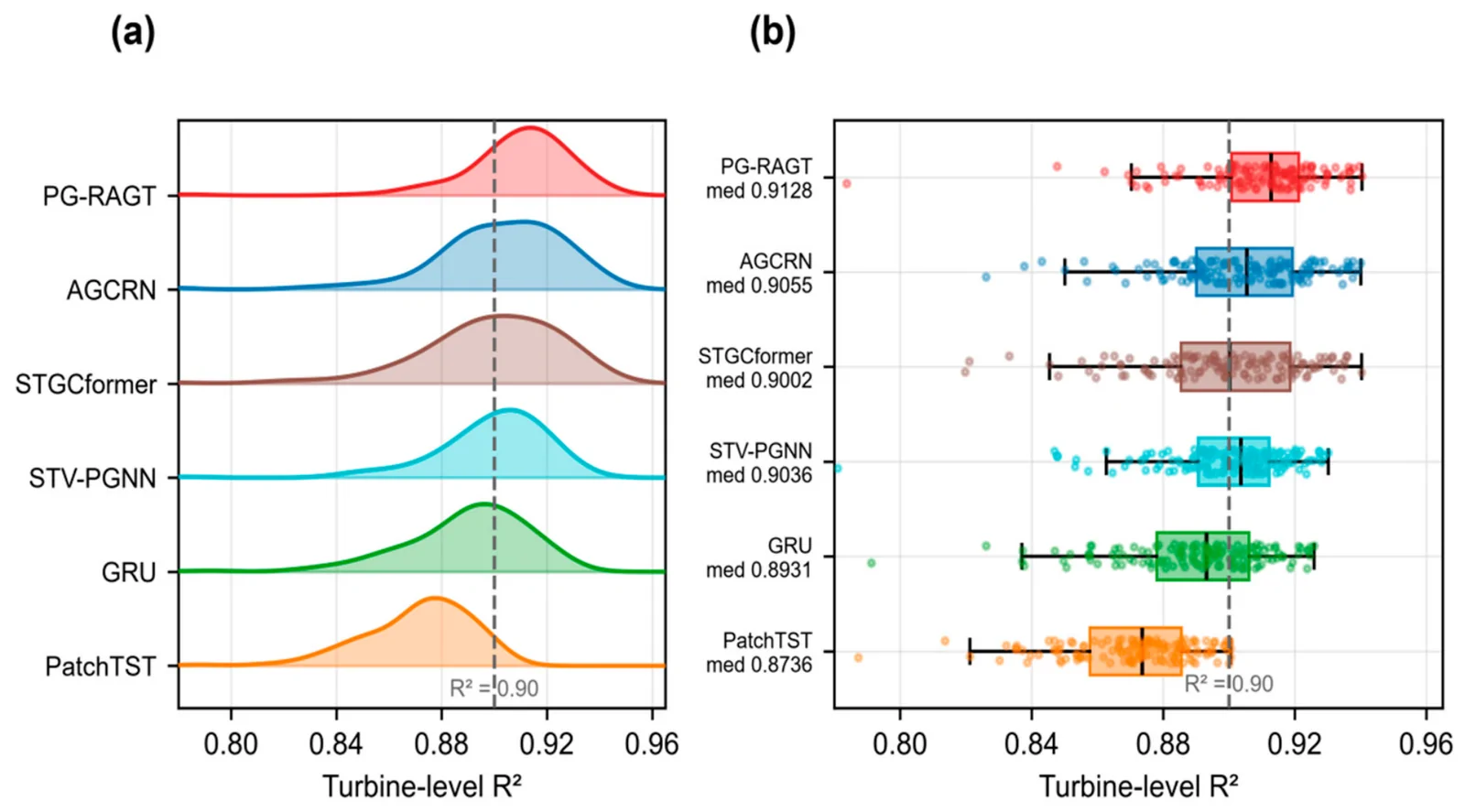
Distribution of turbine-level *R*^2^ across the neural forecasting models: (**a**) density distributions and (**b**) boxplots.

Figure 9 examines the distribution of turbine-level *R*^2^ to determine whether the overall improvement is concentrated in a small number of turbines or distributed more broadly across the wind farm.

As shown in Figure 9, PG-RAGT has the highest median turbine-level *R*^2^ at 0.9128. The corresponding medians are 0.9055 for AGCRN, 0.9036 for STV-PGNN, 0.9002 for STGCformer, 0.8931 for GRU, and 0.8736 for PatchTST. Its turbine-level distribution is also shifted toward higher *R*^2^ values across much of the fleet. From the fleet-level view, the improvement therefore appears to be relatively broad rather than being associated with only a few favorable turbines. This pattern is consistent with the substantial degradation observed when cross-turbine attention is removed in Table 6, although the distribution itself does not identify the individual interactions responsible for the gain.

#### 4.4.4. Prediction-Interval Performance

Table 13 compares the uncertainty output of PG-RAGT with Quantile-PatchTST, our quantile-regression adaptation of the evaluated PatchTST backbone [15], rather than a separately published model. The baseline retains the PatchTST backbone and chronological training protocol, replacing the point head with outputs at q = 0.10, 0.50, and 0.90 trained by masked pinball loss; no post hoc calibration or quantile-order constraint is used. Its output head parameterizes all quantiles as 1.05 × sigmoid(·), so the predicted lower bound cannot equal exactly zero, unlike the zero-clamped lower bound of PG-RAGT.

On the positive-power subset (2,607,038 common valid targets), PG-RAGT and Quantile-PatchTST have comparable empirical PICP for the nominal 80% interval (82.50% versus 81.80%). However, PG-RAGT produces narrower intervals (MPIW: 295.47 versus 394.43 kW; 25.1% lower), lower mean pinball loss (28.53 versus 36.17 kW; 21.1% lower), and a lower interval score (419.10 versus 516.84 kW; 18.9% lower). These subgroup statistics were recomputed from the saved predictions using identical targets and evaluation masks and are included in the Results folder of the Supplementary Materials. For all 4,241,719 valid test targets, PG-RAGT obtains PICP = 88.73%, MPIW = 197.33 kW, mean pinball loss = 18.76 kW, and interval score = 277.34 kW, compared with 50.60%, 278.32 kW, 24.38 kW, and 359.76 kW for Quantile-PatchTST. However, 38.54% of these targets are genuine zero-power observations, and the strictly positive lower-quantile parameterization of Quantile-PatchTST cannot represent an exact zero, unlike PG-RAGT’s zero-clamped lower bound. Thus, the full-sample coverage difference cannot be attributed to calibration alone. The quantile heads are not lower-bound parameterization matched, and the positive-power analysis supports a more favorable empirical coverage–sharpness trade-off under the evaluated configurations, rather than a general claim of superiority.

#### 4.4.5. Screening Behavior

**Figure 10:**
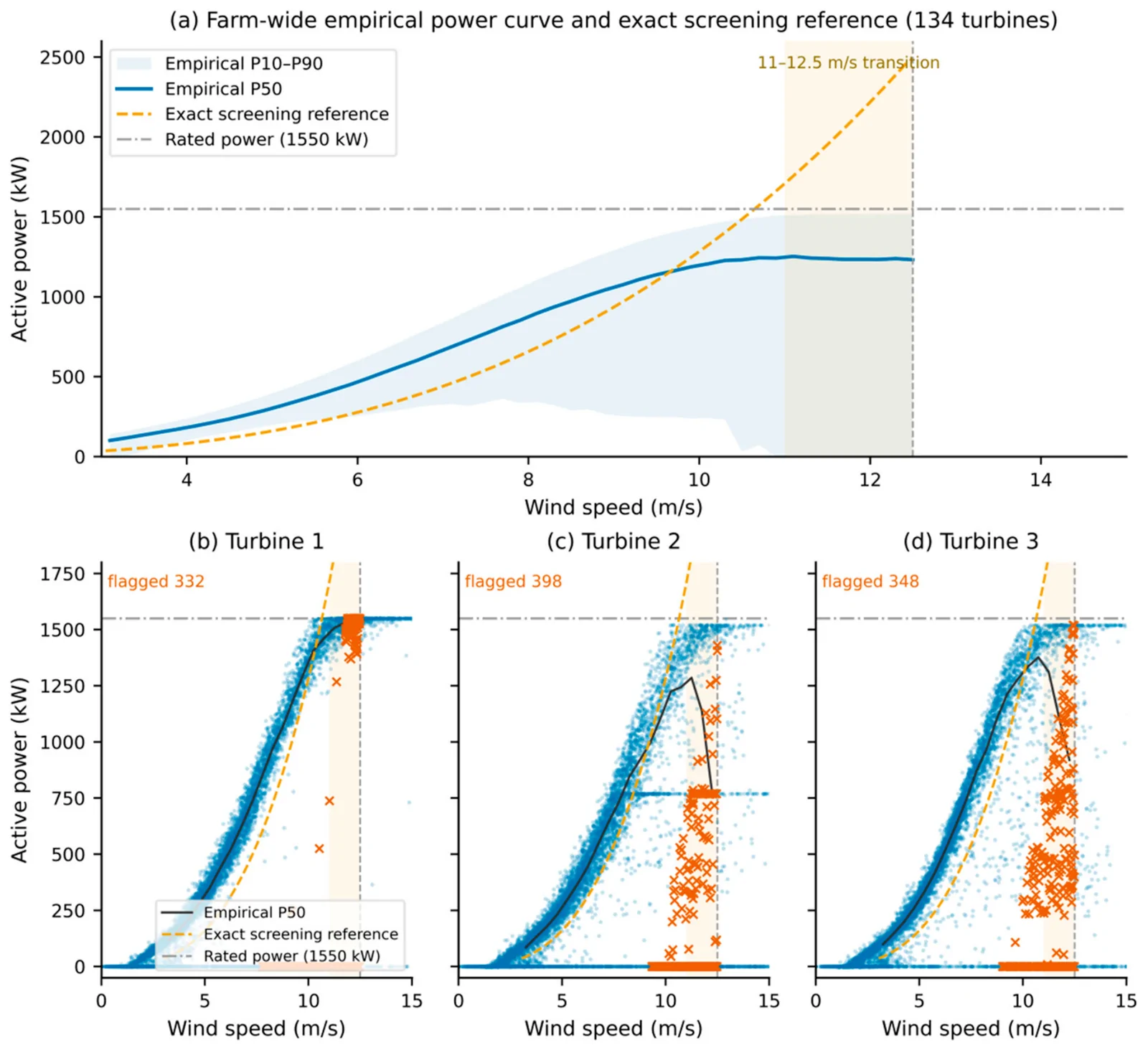
Wind farm screening audit: (**a**) empirical P10–P90 band, P50, and uncapped reference; (**b**–**d**) flagged observations for Turbines 1–3. Shading indicates 11–12.5 m/s; the horizontal line marks rated power (1550 kW). In (b–d), blue dots represent the original SCADA power observations, while orange crosses indicate observations flagged by the screening procedure.

Figure 10 audits Physics-Consistent SCADA Screening against the empirical power behavior, using both the full 134-turbine fleet and the original 30,000-sample subset from Turbines 1–3.

As shown in Figure 10, the original 30,000-sample audit contains 1078 flagged observations (3.59%). Across all 134 turbines, 109,945 of 2,900,809 observations satisfying the screening interval are flagged, corresponding to a farm-wide rate of 3.79%; the per-turbine rates range from 2.98% to 6.27%, with a median of 3.73%. The empirical comparison shows that the nominal reference under-predicts the median power in the lower Partial-Load range but exceeds the empirical median from approximately 9.8 m/s onward, with the largest divergence in the 11–12.5 m/s Transition region because the uncapped reference continues to increase while measured power begins to saturate. Consequently, some valid rated or pitch-regulated observations can be flagged as nominal-reference deviations. Farm-wide, 2.37% of flagged observations have Patv ≥ 1500 kW and 0.30% satisfy the stricter rated-like criterion Patv ≥ 0.99*P*_rated_, whereas 84.75% are below 1000 kW. Rated-like observations have a farm-wide wind speed median of 12.83 m/s (P10 = 11.29 m/s), and the empirical P90 first reaches 98% of rated power at approximately 12.3 m/s; thus, the adopted 12.5 m/s rated-speed parameter is not contradicted by the observed full-power behavior, although it should not be interpreted as a sharp empirical boundary for every turbine. These results support interpreting the screening as a nominal-regime training filter rather than a sensor-fault classifier. The original active-power observations and target-validity mask are preserved for evaluation. Flagged active-power values are imputed for model-input construction, while the corresponding target positions are excluded only from the training loss.

#### 4.4.6. External Wind Farm Validation

To examine applicability under a different wind farm setting, PG-RAGT is retrained and evaluated on the Penmanshiel dataset [33] against PatchTST, GRU, AGCRN, STGCformer, and STV-PGNN using the same chronological protocol. Table 14 summarizes the point-forecasting results.

On Penmanshiel, PG-RAGT gives *R*^2^ = 0.8356, RMSE = 287.93 kW, MAE = 186.36 kW, and MAPE = 33.10%. STGCformer is the closest baseline in RMSE at 291.33 kW, followed by AGCRN at 293.11 kW and STV-PGNN at 294.48 kW. Relative to AGCRN, the RMSE and MAE reductions are approximately 1.8% and 2.5%, which are smaller than on SDWPF. A Diebold–Mariano test based on per-window MAE for 7879 Penmanshiel test windows gives DM = −5.686 and *p* = 1.30 × 10^−8^ for PG-RAGT versus STGCformer. Thus, a statistical difference is detected under this retrained external-site protocol, while the numerical margin remains modest. The experiment demonstrates applicability after retraining rather than zero-shot transfer. Related industrial prognostics work has addressed cross-domain prediction through explicit representation adaptation [38]; no such domain-adaptation mechanism is used here.

## 5. Discussion

### 5.1. Performance Across Forecasting Scenarios and Wind Farms

Across forecast horizons, operating conditions, and turbine units, PG-RAGT shows a broadly consistent advantage, although the size of the gain is not uniform. On SDWPF, the improvement over the parameter-matched AGCRN is more evident in MAE than in RMSE, suggesting that typical absolute errors are reduced more clearly than errors emphasized by the squared-error metric. The fleet-level distribution supports the same interpretation: the median turbine-level *R*^2^ reaches 0.9128 for PG-RAGT and 0.9055 for AGCRN, while STV-PGNN and STGCformer reach 0.9036 and 0.9002, respectively. The PG-RAGT distribution remains shifted toward higher values across much of the fleet.

Forecast horizon has a visible influence on relative performance. PG-RAGT retains lower RMSE than AGCRN at all six horizons, but the gap narrows as the target moves farther from the observed history. Because the six forecasts are generated from a shared historical context with horizon-specific embeddings, this pattern is not caused by autoregressive error propagation. Rather, historical SCADA becomes less informative for farther targets, while the states estimated by Gated Physical-State Rolling are also farther from their common observed origin. From the present results, the advantage of PG-RAGT is therefore comparatively more apparent at shorter forecast horizons.

Operating-condition and external-site results show that the gain depends on the forecasting scenario. PG-RAGT performs best under calm conditions, whereas STGCformer has the lowest RMSE and MAE during ramp events. Partial Load is the strongest operating regime for PG-RAGT; at Rated Operation, persistence remains slightly better and STGCformer is also marginally lower in RMSE. On Penmanshiel, PG-RAGT retains the lowest aggregate RMSE after retraining, but the margin over the strongest baselines is smaller than on SDWPF. The nominal 80% interval has 88.73% coverage overall, but 38.54% of the valid samples are measured zero-output observations; on positive-power samples, coverage is 82.50% for PG-RAGT and 81.80% for Quantile-PatchTST. PG-RAGT retains narrower intervals and lower pinball and interval scores on these operating samples, although the models use different lower-quantile output bounds. The model should therefore be viewed as comparatively accurate across the evaluated settings rather than uniformly best under every operating condition.

### 5.2. Comparison with Existing Methods and Component Contributions

The comparison with existing forecasting methods suggests that the gain of PG-RAGT is related more to the task-specific organization of the model than to parameter count alone. GRU and PatchTST provide temporal references, AGCRN additionally models inter-turbine dependence, while STGCformer and STV-PGNN provide recent graph Transformer and physics-guided spatiotemporal graph references. The trainable models contain approximately 210–229-thousand parameters, and PG-RAGT nevertheless gives the lowest aggregate forecasting errors on SDWPF. A closer comparison with AGCRN remains particularly informative. Its adaptive adjacency is shared across input samples in the implemented baseline, whereas PG-RAGT recomputes cross-turbine attention weights from the current patch representations. The contribution claimed here is therefore not adaptive graph learning itself, but the state-conditioned fleet interaction formulation within the coupled SCADA forecasting architecture.

Recent attention-based diagnostic work has supplemented attention and fusion modules with explicit interpretability analysis to quantify learned branch contributions [39]. Motivated by the same need for empirical interpretation, a post hoc audit of the last Axial Fleet Block was performed over all 5287 SDWPF test windows without retraining. The mean normalized Frobenius change between adjacent temporal patches is 0.593, while the mean deviation from the patch-wise test-set mean interaction matrix is 0.632; the median cosine similarity to that mean is 0.927. These values confirm patch- and sample-dependent variation while retaining a shared interaction pattern. By contrast, the association between mean attention and turbine coordinate distance is weak (Spearman rho = −0.033). The five highest-attention non-self neighbors are on average about 12% closer in the provided coordinate system than the complete set of candidate neighbors, but the coordinate units and wind-direction convention could not be verified sufficiently for a defensible wake-alignment analysis. The audit therefore supports interpreting the attention matrix as a dynamic learned fleet interaction representation, not as a recovered physical wake graph.

The multi-seed ablation results sharpen this interpretation. Cross-turbine attention is the most stable accuracy contributor, with a paired RMSE degradation of 8.80 ± 2.25 kW when removed. By comparison, the mean paired effects are 2.06 ± 2.25 kW for Physics-Consistent SCADA Screening and 0.80 ± 1.27 kW for Gated Physical-State Rolling. The smaller components are training-run sensitive, so they are not presented as uniformly improving RMSE. The controlled AGCRN preprocessing check reaches a similar conclusion: disabling Physics-Consistent SCADA Screening changes AGCRN RMSE by only +0.26 kW and MAE by −0.35 kW. This indicates a small and model-dependent preprocessing effect rather than a general source of baseline improvement.

Gated Physical-State Rolling is better interpreted as a forecast-time physical-state closure mechanism than as the main accuracy driver. It generates explicit horizon-dependent wind speed, external temperature, and mean pitch angle without reading future SCADA, which keeps the aerodynamic prior and its associated physics-consistency guidance horizon-specific over all six horizons. Across the three seeds, removal of the rolling pathway increases MAE by 1.13 ± 0.53 kW on average, while the RMSE effect is smaller and includes one modest reversal. The gate audit provides an additional boundary on the claim: the direct physical-candidate weight averages only 0.29%, whereas the final mixture is dominated by persistence and the data expert. Physical guidance therefore acts mainly through rolled-state supervision and the horizon-specific Partial-Load physics-consistency regularizer, not through a large direct physical expert weight.

The additional modeling stages introduce a computational trade-off, but the measured cost remains small relative to the 10 min SCADA interval. The full model contains 219,809 trainable parameters and requires about 0.87 ms per full-farm forecasting window for the forward pass on the RTX 4050 Laptop GPU. A one-block variant reduces the parameter count by 26.6% and latency by 17.7%, while RMSE increases by only about 0.45% on seed 2026. This reduced configuration does not replace the two-block model used for the main results, but it shows that lower-cost deployment is possible without a large RMSE penalty.

### 5.3. Limitations and Future Work

Several limitations remain. First, the screening rule and the Differentiable Wind Power Prior use simplified aerodynamic assumptions. In the upper Transition region, the uncapped screening reference can exceed the empirical operating curve as power begins to saturate, so some valid pitch-regulated or power-limited observations may be flagged. The fixed *λ*_TSR_ = 7.5 and the nominal screening coefficient *C_p_* = 0.45 are also approximations rather than turbine-specific operating optima. For offshore farms or newer turbines with larger rotors, higher rated power, different rated wind speeds, and stronger wake interactions, the screening interval, rotor radius, rated-speed parameters, and prior calibration should be re-estimated from the corresponding training data before application.

Second, the accuracy advantage is not uniform across forecast horizons or operating conditions. The margin narrows at longer horizons, STGCformer performs better during ramp events, and persistence remains slightly better in Rated Operation. The external-site gain after retraining is also smaller than on SDWPF. The probabilistic output is supported by one quantile-regression baseline, but broader calibration studies and comparisons with additional probabilistic methods remain outside the present scope.

Finally, the fleet-level interaction model has approximately quadratic cross-turbine cost at each temporal patch. The present experiments cover 14- and 134-turbine farms, while substantially larger arrays may require sparsified or more efficient interaction mechanisms. The attention audit also shows only a weak global association with turbine coordinate distance; because the SDWPF coordinate unit and wind-direction convention cannot be verified well enough for directional wake reconstruction, the attention matrix is treated as a state-conditioned statistical fleet representation rather than as a recovered physical wake graph. Cross-domain generalization is likewise not claimed, since the Penmanshiel model is retrained for that site.

## 6. Conclusions

This study develops PG-RAGT as an application-oriented system for fleet-scale short-term wind power forecasting from SCADA data, with state-conditioned fleet interaction as the main architectural contribution and Gated Physical-State Rolling as the main methodological contribution; Physics-Consistent SCADA Screening remains a supporting nominal-regime training filter. The main seed on SDWPF achieves *R*^2^ = 0.9125, RMSE = 106.69 kW, and MAE = 57.12 kW; across three independently seeded runs, the mean RMSE is 107.19 ± 1.17 kW. Relative to persistence, the main run gives RMSE and MAE skill scores of 10.15% and 6.21%. External-site validation on Penmanshiel shows that the framework remains applicable after retraining, with a smaller numerical margin than on SDWPF. The conclusions below are therefore limited to the evaluated SCADA settings and do not imply uniform superiority under every operating condition.

(1) Cross-turbine attention provides the strongest and most stable component-level accuracy contribution. Removing the cross-turbine pathway increases RMSE by 8.80 ± 2.25 kW across the three seeds. The post hoc audit confirms dynamic variation in the learned matrices, while the weak global distance association indicates that they should not be interpreted as deterministic wake graphs.
(2) Gated Physical-State Rolling provides the task-specific forecast-time physical-state closure capability. Bounded cumulative updates from a common observed origin generate horizon-dependent wind speed, external temperature, and mean pitch angle, so the state-dependent aerodynamic prior can be evaluated without future SCADA measurements. Its direct RMSE contribution is modest and training-run dependent (paired ΔRMSE = 0.80 ± 1.27 kW), while the paired MAE contribution is more consistent (1.13 ± 0.53 kW). The direct physical-candidate fusion weight is small, so the physical pathway is interpreted mainly as auxiliary state and physics guidance during joint training.
(3) Supporting evaluations define the practical scope of the framework. Physics-Consistent SCADA Screening has a mean paired RMSE effect of 2.06 ± 2.25 kW and is treated as a nominal-regime filter rather than a sensor-fault detector. PG-RAGT has lower pinball loss and interval scores than the evaluated Quantile-PatchTST baseline, including on positive-power samples, although the full-sample coverage difference is affected by unequal lower-quantile parameterizations. The one-block variant reduces parameters by 26.6% and inference latency by 17.7% with an RMSE increase of about 0.45%. The model is not best during ramp events or Rated Operation, and higher-wind or offshore applications would require recalibration of the screening and aerodynamic-prior parameters.

## Figures and Tables

**Table 1:** Sentinel-value flags by measurement channel (shares below 0.01% are shown to three decimal places).

Channel	Flags	Share
Etmp	271,060	5.73%
Itmp	11,908	0.25%
Wdir	78	0.002%
Pab1-3	135	0.003%
Wspd/Prtv	0	0.00%

**Table 2:** Baseline models, trainable parameters, and model-fitting times.

Model	Trainable Parameters	Time (min)	Comparison Role
Persistence	0	0.0	Last-value baseline
GRU	219,342	19.6	Recurrent temporal baseline (d = 152, parameter-matched)
PatchTST	210,342	11.0	Patch-based Transformer baseline
AGCRN	220,464	36.0	Adaptive graph baseline (d = 174, parameter-matched)
STGCformer	225,783	18.0	Spatiotemporal graph convolutional Transformer baseline (reimplemented)
STV-PGNN	228,816	12.0	Wake-physics GNN baseline (reimplemented)
Aerodynamic Prior	402	0.0	Aerodynamic-prior baseline
PG-RAGT	219,809	11.6	Proposed model

**Table 3:** Horizon-wise physical-state prediction MAE.

Step Size	Wind Speed MAE (m/s)	External-Temperature MAE (°C)	Mean-Pitch-Angle MAE (°)
10 min	0.303	0.199	2.622
20 min	0.428	0.346	4.313
30 min	0.519	0.475	5.738
40 min	0.595	0.595	7.047
50 min	0.661	0.708	8.279
60 min	0.720	0.816	9.463

**Table 4:** Distribution of the state-update gate values by operating regime.

Operating Regime	Samples	Wind Speed Gate	External-Temperature Gate	Mean-Pitch-Angle Gate
Shutdown/Low Output	2,228,634	0.124	0.103	0.060
Partial Load	1,838,124	0.191	0.189	0.031
Rated Operation	183,990	0.203	0.146	0.041
Overall	4,250,748	0.157	0.142	0.047

**Table 5:** Training-loss components at the first and final epochs.

Loss Term	Initial	Final	Change
*L*_point_	0.0753	0.0417	−45%
*L*_quantile_	0.0363	0.0189	−48%
*L*_state_	0.1216	0.0952	−22%
*L*_regime_	0.7945	0.4012	−50%
*L*_ramp_	0.0443	0.0438	−1%
*L*_physics_	0.1686	0.0733	−57%

**Table 6:** Three-seed SDWPF ablation results (mean ± SD; paired changes are ablation minus full model).

Variant	RMSE (kW)	*R* ^2^	MAE (kW)	Paired ΔRMSE (kW)	Paired ΔMAE (kW)
Full PG-RAGT	107.19 ± 1.17	0.9116 ± 0.0020	57.42 ± 0.50	--	--
w/o Cross-Turbine Attention	115.99 ± 1.37	0.8965 ± 0.0025	62.38 ± 0.83	+8.80 ± 2.25	+4.96 ± 1.28
w/o Physics-Consistent SCADA Screening	109.25 ± 1.26	0.9082 ± 0.0021	59.25 ± 1.53	+2.06 ± 2.25	+1.83 ± 1.92
w/o Gated Physical-State Rolling	107.99 ± 1.90	0.9103 ± 0.0032	58.55 ± 0.73	+0.80 ± 1.27	+1.13 ± 0.53

**Table 7:** Diebold–Mariano test results for the main seed 2026.

Comparison	DM Statistic	*p*-Value	ΔMAE (Ablation − Full) (kW)	95% CI of ΔMAE (kW)
w/o Cross-Turbine Attention	−9.20	<0.001	5.28	[3.86, 6.83]
w/o Physics-Consistent SCADA Screening	−6.86	<0.001	1.77	[1.16, 2.38]
w/o Gated Physical-State Rolling	−3.84	0.0001	1.65	[0.62, 2.79]

**Table 8:** Deterministic forecasting performance on the SDWPF test set.

Model	RMSE (kW) ↓	*R*^2^ ↑	MAE (kW) ↓	MAPE ↓	Parameters
Persistence	118.75	0.8916	60.90	31.15%	0
GRU	117.10	0.8946	67.43	32.13%	219,342
PatchTST	127.14	0.8757	76.09	37.45%	210,342
AGCRN	109.87	0.9072	63.96	27.91%	220,464
STGCformer	111.74	0.9040	66.25	30.92%	225,783
STV-PGNN	112.75	0.9022	63.28	28.02%	228,816
Aerodynamic Prior	179.60	0.7520	103.45	51.82%	402
PG-RAGT	106.69	0.9125	57.12	27.09%	219,809

Note: ↑ indicates that higher values are better, whereas ↓ indicates that lower values are better.

**Table 9:** Three-seed robustness on SDWPF (mean ± SD).

Model	Seed	RMSE (kW)	*R* ^2^	MAE (kW)	MAPE (%)
PG-RAGT	2025	106.35	0.9130	57.15	26.52
	2026	106.69	0.9125	57.12	27.09
	2027	108.53	0.9094	57.99	28.11
	Mean ± SD	107.19 ± 1.17	0.9116 ± 0.0020	57.42 ± 0.50	27.24 ± 0.81
AGCRN	2025	110.14	0.9067	67.73	28.12
	2026	109.87	0.9072	63.96	27.91
	2027	109.10	0.9085	64.04	27.98
	Mean ± SD	109.70 ± 0.54	0.9075 ± 0.0009	65.24 ± 2.15	28.00 ± 0.11
STGCformer	2025	111.85	0.9038	68.23	28.62
	2026	111.74	0.9040	66.25	30.92
	2027	112.54	0.9026	65.63	29.87
	Mean ± SD	112.04 ± 0.43	0.9035 ± 0.0008	66.70 ± 1.36	29.80 ± 1.15
STV-PGNN	2025	112.71	0.9023	66.44	29.21
	2026	112.75	0.9022	63.28	28.02
	2027	113.29	0.9013	61.93	27.84
	Mean ± SD	112.92 ± 0.32	0.9019 ± 0.0006	63.88 ± 2.31	28.36 ± 0.74
GRU	2025	119.81	0.8896	70.43	29.75
	2026	117.10	0.8946	67.43	32.13
	2027	116.86	0.8950	69.95	31.28
	Mean ± SD	117.92 ± 1.64	0.8931 ± 0.0030	69.27 ± 1.61	31.05 ± 1.21
PatchTST	2025	134.22	0.8615	88.42	42.35
	2026	127.14	0.8757	76.09	37.45
	2027	130.57	0.8689	80.52	36.64
	Mean ± SD	130.64 ± 3.54	0.8687 ± 0.0071	81.68 ± 6.25	38.81 ± 3.09

**Table 10:** Horizon-wise RMSE and MAE for PG-RAGT and AGCRN on SDWPF (seed 2026).

Horizon	PG-RAGT RMSE	AGCRN RMSE	RMSE Reduction	PG-RAGT MAE	AGCRN MAE	MAE Reduction
10 min	63.47	69.47	8.6%	31.86	39.87	20.1%
20 min	84.84	88.56	4.2%	44.77	51.12	12.4%
30 min	100.26	101.88	1.6%	54.52	60.13	9.3%
40 min	113.51	115.37	1.6%	62.99	68.66	8.2%
50 min	125.22	127.72	2.0%	70.73	77.82	9.1%
60 min	135.85	140.55	3.3%	77.85	86.17	9.7%
Overall	106.69	109.87	2.9%	57.12	63.96	10.7%

**Table 11:** RMSE/MAE (kW) under calm and ramp conditions on the SDWPF test set.

Model	Calm RMSE	Calm MAE	Ramp RMSE	Ramp MAE
Persistence	103.35	53.54	298.07	221.74
GRU	103.09	60.44	284.89	220.10
PatchTST	114.29	69.23	289.78	226.00
AGCRN	96.17	57.27	271.57	210.15
STGCformer	99.22	59.94	264.95	204.27
STV-PGNN	98.44	56.32	280.71	215.49
PG-RAGT	92.26	50.25	272.28	207.34

**Table 12:** RMSE (kW) and *R*^2^ by operating regime.

Model	Shutdown/Low Output	Partial Load	Rated Operation
Persistence	45.56 (*R*^2^ −0.644)	161.57 (*R*^2^ 0.728)	199.07 (*R*^2^ 0.847)
GRU	45.79 (*R*^2^ −0.660)	157.66 (*R*^2^ 0.741)	207.06 (*R*^2^ 0.835)
PatchTST	62.57 (*R*^2^ −2.100)	162.70 (*R*^2^ 0.724)	247.62 (*R*^2^ 0.763)
AGCRN	45.32 (*R*^2^ −0.626)	143.60 (*R*^2^ 0.785)	218.61 (*R*^2^ 0.816)
STGCformer	56.28 (*R*^2^ −1.508)	144.83 (*R*^2^ 0.781)	200.90 (*R*^2^ 0.844)
STV-PGNN	51.54 (*R*^2^ −1.103)	146.89 (*R*^2^ 0.775)	214.00 (*R*^2^ 0.823)
Aerodynamic Prior	40.06 (*R*^2^ −0.271)	246.88 (*R*^2^ 0.364)	341.06 (*R*^2^ 0.551)
PG-RAGT	44.33 (*R*^2^ −0.556)	140.82 (*R*^2^ 0.793)	202.25 (*R*^2^ 0.842)

**Table 13:** Nominal 80% prediction-interval results on SDWPF for all valid targets and positive-power targets (seed 2026).

Model	PICP ↑	MPIW (kW) ↓	PINAW ↓	Mean Pinball (kW) ↓	Interval Score (kW) ↓
PG-RAGT	88.73%	197.33	0.1273	18.76	277.34
Quantile-PatchTST	50.60%	278.32	0.1796	24.38	359.76
PG-RAGT (positive power)	82.50%	295.47	0.1906	28.53	419.10
Quantile-PatchTST (positive power)	81.80%	394.43	0.2545	36.17	516.84

Note: ↑ indicates that higher values are better, whereas ↓ indicates that lower values are better.

**Table 14:** External-site results on the Penmanshiel test segment after retraining.

Model	RMSE (kW)	*R* ^2^	MAE (kW)	MAPE
PatchTST	297.43	0.8246	193.79	34.80%
GRU	293.63	0.8290	190.10	33.70%
AGCRN	293.11	0.8296	191.18	33.70%
STGCformer	291.33	0.8317	194.20	33.79%
STV-PGNN	294.48	0.8280	195.05	34.28%
PG-RAGT	287.93	0.8356	186.36	33.10%

## Data Availability

The SDWPF dataset [25] (https://aistudio.baidu.com/competition/detail/152/0/introduction; accessed on 13 July 2026) used in this study is publicly available. The Penmanshiel dataset [33] is publicly available on Zenodo (https://doi.org/10.5281/zenodo.5946808; accessed on 5 August 2026). File S1 contains the PG-RAGT source code and 14 supporting CSV results; main-text figures are supplied separately as production files.

## Supplementary Materials

The following supporting information can be downloaded at https://www.mdpi.com/article/10.3390/s26216672/s1. File S1: PG-RAGT source code, experiment configurations, and supporting numerical results (ZIP archive).

## Abbreviations

SCADA, Supervisory Control and Data Acquisition; PG-RAGT, the proposed physics-guided graph Transformer; LSTM, Long Short-Term Memory; GRU, Gated Recurrent Unit; GCN, Graph Convolutional Network; GAT, Graph Attention Network; AGCRN, Adaptive Graph Convolutional Recurrent Network; RMSE, root mean square error; MAE, mean absolute error; MAPE, mean absolute percentage error; PICP, prediction interval coverage probability; MPIW, mean prediction interval width; PINAW, prediction interval normalized average width; IQR, interquartile range; DM, Diebold–Mariano; CI, confidence interval; GPU, graphics processing unit.

## Nomenclature

*P*_rated_, adopted site-specific power reference; Paero, pre-transition aerodynamic power (kW); Pphys, normalized output of the Differentiable Wind Power Prior; *P*_data_, data-expert output; *P*_pers_, persistence-expert output; *C_p_*, power coefficient; *λ*_TSR_, tip-speed ratio used by the aerodynamic prior; *β*_pitch_, mean blade-pitch angle; *z_h_*, horizon-wise latent representation; *s_h_*, predicted physical state at horizon *h*; Δ*s_j_*, bounded normalized state increment; *g_j_*, state-update gate; *ρ*, air density; *R*, rotor radius; *η*, electromechanical efficiency.
